# Investigating Alzheimer’s Disease-Associated Genes Using Differential Splicing Frequency Analysis

**DOI:** 10.3390/cells15121086

**Published:** 2026-06-15

**Authors:** Yang Yao, Sha Zhou, Zhi Cheng, Shunmei Chen, Yiyao Zhang, Jingsong Shi, Dongsheng Wei, Tao Zhang, Guangyou Duan, Shan Gao

**Affiliations:** 1 Department of Neurology, Tianjin First Central Hospital, Nankai University, Tianjin 300192, China; 2 College of Life Sciences, Nankai University, Tianjin 300071, China; 3 State Key Laboratory of Medicinal Chemical Biology, College of Chemistry, Nankai University, Tianjin 300071, China; 4 Biomedical Engineering Research Institute, Kunming Medical University, Kunming 650500, China; 5 National Clinical Research Center for Kidney Diseases, Affiliated Jinling Hospital, Medical School, Nanjing University, Nanjing 210016, China; 6 School of Life Sciences, Qilu Normal University, Jinan 250200, China

**Keywords:** alternative splicing, splice junction usage, transcriptome analysis, DGEA, *APOE*

## Abstract

Accurately quantifying the expression of individual transcript isoforms remains a formidable challenge, especially in contexts such as neurodegenerative diseases and cancers, which are characterized by high isoform diversity. The present study introduces a junction-based method, named differential splicing frequency analysis (DSFA), which enables more sensitive detection of differential splicing using RNA-seq data. Unlike the existing exon-, isoform-, and event-based methods, DSFA quantifies splice junction usage. We applied DSFA to Alzheimer’s disease (AD)-associated genes through large-scale RNA-seq data mining. The present study is the first to establish that the APP770-, APP751-, APP695-, and APP752-encoding isoforms represent major isoforms of the *APP* gene. Three important findings are: (1) the APP752-encoding isoform exhibits immune cell specificity; (2) the relative proportion of the APP752-encoding isoform increases during the differentiation of induced pluripotent stem cells (iPSCs) into microglia, akin to the increase in relative proportion of the APP695-encoding isoform during iPSC differentiation into neurons; and (3) the APP751-encoding isoform predominates in both cancer and immune cells. Additionally, we identified APP/58417N and App/52804N as differentially expressed splice junctions in humans and mice, respectively. Through over-expression of U1 snRNA in human embryonic stem cell (hESC)-derived neurons, we found that U1 snRNA over-expression decreases the usage of APP/58417N in neurons, similar to the effects observed in AD samples. Our research highlights that the major isoforms of a gene can differ markedly in their expression across tissue and cell types.

## 1. Introduction

RNA sequencing (RNA-seq), a high-throughput gene expression measurement technology, is widely utilized in life sciences for its comprehensive view of the transcriptome. The transcriptome is traditionally defined as encompassing mRNAs and long non-coding RNAs (lncRNAs) exceeding 200 nucleotides in length. RNA-seq outperforms microarray technologies in specificity, enabling better discrimination among isoforms. However, many researchers still treat genes as single units, ignoring isoform-level differences, when quantifying gene expression using RNA-seq data, particularly in differential gene expression analysis (DGEA) [1]. This may inadvertently neglect the substantial isoform-level changes even when overall gene expression remains unchanged. On the other hand, other researchers employ advanced bioinformatics tools to quantify individual isoform expression. These tools rely on predictive models rather than direct quantification, because RNA-seq reads are short. The predictive models use algorithms to allocate reads to the most probable isoforms, taking into account various factors such as read coverage and known transcript structures. A previous study [2] concluded that none of the evaluated methods is accurate enough for de novo transcriptome assembly, where isoform structures must be inferred directly from the RNA-Seq data. Thus, accurate isoform quantification remains challenging, especially in contexts like neurodegenerative diseases [3] and cancers [4], where isoform diversity is high.

Alternative technologies have been developed to improve the quantification of individual isoforms, addressing challenges posed by high isoform diversity. Tiling array [5] and HIDE-seq [6], as such technologies, have been employed to achieve isoform-level resolution across the genome. More promising are long-read sequencing platforms, including Pacific Biosciences’ full-length transcriptome sequencing (PacBio cDNA-seq) [7] and Oxford Nanopore Technologies’ direct RNA sequencing (Nanopore RNA-seq) [8]. These platforms produce long reads that can, in theory, quantify nearly all isoforms of a gene. However, they remain limited by lower throughput, reduced sequence fidelity, and higher costs [9]. They also present a new challenge: the increasing discovery of novel splice junctions and isoforms in certain genes. For example, a recent study focusing on tau pathology [10] identified 1408 isoforms in *APOE* alone—only 13 previously known and 1395 newly discovered. Consequently, most transcriptome sequencing still relies on short-read technologies—a trend likely to continue until long-read technologies mature. Rather than adopting alternative technologies, developing computational methods to fully exploit differential splicing information already present in short-read RNA-seq data is still a highly promising strategy.

To more sensitively detect differential splicing, researchers have developed exon-based (e.g., JunctionSeq), isoform-based (e.g., Cuffdiff2 and DiffSplice) and event-based methods (e.g., MAJIQ, rMATS, SUPPA, and dSpliceType) [11]. Yet, to the best of our knowledge, none of these methods has been leveraged to pinpoint a specific splicing event significantly associated with AD onset or progression. We therefore developed a junction-based method that first quantifies the expression levels of splice junctions and then identifies splice junctions whose usage differs significantly between two or more groups or conditions; these are referred to as significantly differentially expressed (DE) splice junctions. As DE splice junctions are analogous to DE genes identified by the conventional DGEA, the new method was named differential splicing frequency analysis (DSFA). DGEA focuses specifically on the identification of DE genes from all studied genes, whereas DSFA focuses specifically on the identification of DE splice junctions within each gene. We then applied DSFA to our AD research to both demonstrate its utility and identify DE junctions between AD samples and matched controls or wild-type samples. As part of our AD research, the present study began by applying DSFA to public RNA-seq datasets, focusing on six AD-associated genes (*APP*, *MAPT*, *PSEN1*, *PSEN2*, *APOE*, and *CLU*). The AD cohorts in these RNA-seq datasets were composed chiefly of neuron-, microglia-, and hippocampus-derived samples from AD patients or transgenic mouse models. We then used PacBio cDNA-seq data to confirm DSFA results and validate its reliability.

## 2. Materials and Methods

### 2.1. Data Acquisition and Analysis

By searching “Alzheimer’s Disease” and “RNA-seq”, information of 118 human and 187 mouse projects (Table S1) was retrieved. A total of 117 bioprojects (44 human and 73 mouse) were selected for the re-analysis, based on the following criteria: (1) the exclusion of single-cell RNA-seq (scRNA-seq) and single-nucleus RNA-seq (snRNA-seq) datasets, as well as other non-bulk RNA-seq datasets; (2) the exclusion of datasets associated with irrelevant research topic, e.g., cancer; and (3) the requirement that the average SRA file size (“Bytes”) in a bioproject be above 2 gibibytes (Gb). The workflows of DSFA and DGEA are detailed in Figure S1. DGEA began with the gene expression matrices (read counts) of 117 bioprojects downloaded from the NCBI GEO database. With mitochondria expression data removed, each gene expression matrix was normalized by scaling the raw counts to counts per million (CPM) to identify the significantly DE genes between the AD and control groups, using the R package edgeR [12] v3.22.5. To implement DSFA, Python and Perl scripts are included in Fastq_clean (https://github.com/gaoshanT/Fastq_clean (accessed on 6 June 2026)). Fastq_clean is highly recommended for cleaning and quality control of raw reads, as contaminations such as low-quality nucleotides or even very short residual adapter sequences can result in false-positive counts or splice junctions. DSFA began with the raw data (sequencing reads) of 117 bioprojects downloaded from the NCBI SRA database. The raw data were cleaned using the pipeline Fastq_clean v2.0, and then aligned to the human genome GRCh38/hg38 and mouse genome GRCm39/mm39 using STAR [13] v2.5.2b, respectively. For each non-DE gene, a junction-read-count (JRC) matrix was generated using SAM files derived by STAR, and then row-sum-normalized to identify DE splice junctions between the AD and control groups using the R package edgeR. The criteria for the identification of DE genes are |log2foldchange| > 1 and *p* value < 0.01, whereas those for the DE splice junctions are |log2foldchange| > 0.2 and *p* value < 0.05. The JRC matrices of six AD-associated genes derived from selected RNA-seq datasets and their DSFA results are provided in Supplementary File S2. Statistics and plotting were conducted using the software R v4.3.2 with the Bioconductor packages. The raw HIDE-seq data were downloaded from the website (https://www.med.upenn.edu/dreyfusslab/protocols.html (accessed on 10 June 2024)) and analyzed on a local server. PacBio cDNA data were downloaded from the Pacific Biosciences website, including a dataset from 115 human brain, heart, and liver tissue samples and another dataset from 28 MCF-7 cell samples (Table S2). The primary results from PacBio cDNA data analysis are provided in Supplementary File S3.

### 2.2. Cellular Experiments

The STEMdiff™ Neural System (STEMCELL, Cambridge, MA, USA) was employed to generate hESC-derived neurons according to the manufacturer’s protocol. H1 (WA01) hESCs (RRID: CVCL_9771), maintained in the laboratory, were induced for their differentiation into neural progenitor cells (NPCs) using STEMdiff™ Neural Induction Medium (CATALOG 05835) and a STEMdiff™ Neuron Differentiation Kit (CATALOG 08500). Subsequently, these NPCs were matured into neurons using a STEMdiff™ Neuron Maturation Kit (CATALOG 08510). Full experimental details have been published previously [14]. The identity of these neurons was validated by the expression of *TUJ*, as detected using RT-qPCR. However, the markers *SOX2* and *OCT4* were also detected at moderate levels using RT-qPCR, suggesting the presence of a certain portion of undifferentiated hESCs or NPCs. For each sample, total RNA was isolated using TRIzol Reagent (Thermo Fisher Scientific, Waltham, MA, USA) and the cDNA was synthesized using PrimeScrip RT Reagent Kit (TAKARA, Kusatsu, Japan) and 6-mer random primers. To amplify each gene, 200 ng cDNA was used in qPCR reactions performed on a qPCR instrument LightCycler96 (Roche, Basel, Switzerland) with reaction mixture 2× Super Universal SYBR Master Mix (CWBIO, Taizhou, China). RT-qPCR was also used to confirm the decrease in the usage of APP/58417N upon U1 snRNA over-expression with specific PCR primers (Table S3).

Two groups of hESC-derived neurons were used in the experiments: the U1 group over-expressing U1 snRNA, and the control group. Specifically, two samples of the U1 group, each comprising approximately 1 × 10^6^ cells, were individually transfected with 50 µL lentivirus solution harboring the U1 snRNA expression plasmids, while the two samples of the control group were transfected with lentivirus solution harboring empty plasmids containing 5-bp polyA sequences. The U1 snRNA expression plasmids were constructed by cloning U1 DNA fragments (comprising the native promoter, coding region and terminator of U1 snRNA) into the pLVX-shRNA1 vectors. The coding region of human U1 snRNA is a 164-bp sequence (RefSeq: NR_004430.2). The lentivirus solution was prepared using the Lenti-X™ HTX Packaging System (Clontech, San Jose, CA, USA) and the viral titers were adjusted to 10^8^ infectious units/mL using Lentivirus Concentration Solution (YEASEN, Shanghai, China). Full experimental details for lentivirus transfection and cell culture have been published previously [14]. Total RNA was extracted separately from cells in each sample for library preparation and sequencing on an Illumina HiSeq 2000 sequencer (Illumina, Inc., San Diego, CA, USA). RNA-seq data generated from four samples are publicly available (Data Availability Statement), and the DGEA and DSFA results are provided in Table S4 and Table 1, respectively.

## 3. Results

### 3.1. Differential Splicing Frequency Analysis and APP Isoforms

**Figure 1:**
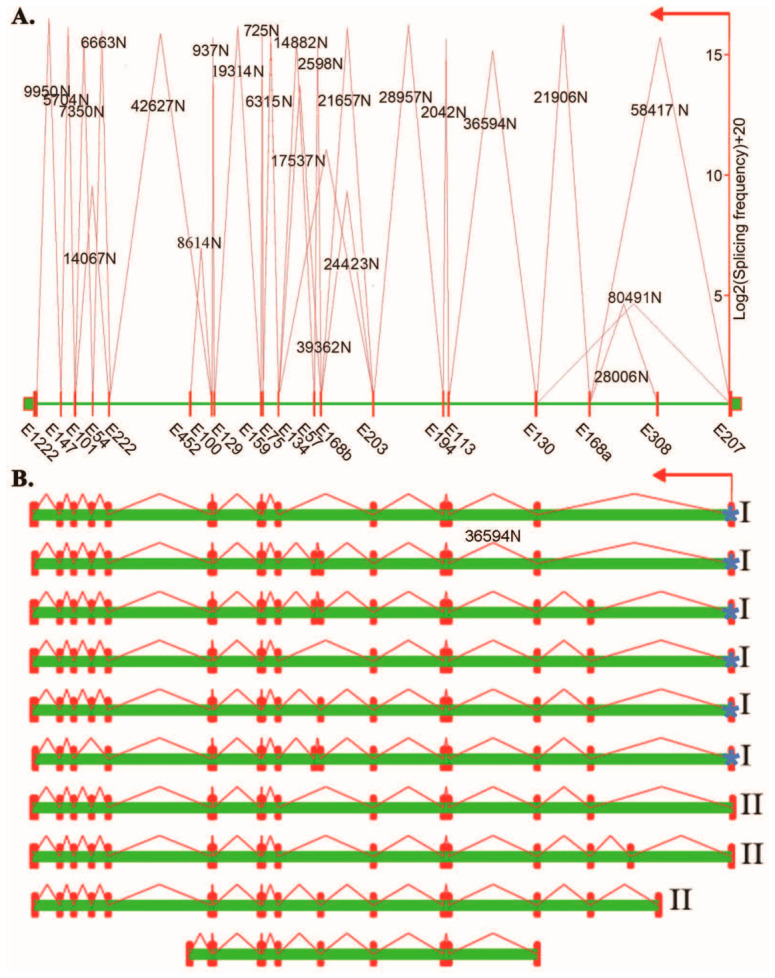
Illustration of the new naming system. The gene orientation matches its genomic orientation, as shown in the genome browser with transcription direction indicated by a red arrow. Exons and introns are indicated in red and green blocks, respectively, while splice junctions are indicated by red lines. Exons and introns in (**A**) were aligned to those in (**B**) for comparison. (**A**) In the new naming system, a splice junction is designated using the name format Gene/yN, where y is the size of its corresponding intron. The normalized expression level of each splice junction is represented by the height of the zigzag line (in red color) above the intron region, which can also be interpreted as the relative frequency of splicing events, termed as junction frequency or usage. The *y*-axis represents the log value of relative frequency plus 20. In total, 24 known splice junctions (Table 1) of the *APP* gene have been quantified using RNA-seq data (SRA: SRR8433687). Among the 24 splice junctions, 5 (APP/80491N, APP/28006N, APP/24423N, APP/8614N, and APP/14067N) are considered low-frequency junctions, as their values fall below 10 on the *y*-axis. Most alternative splicing events occur in the region spanning E134 to E203 (Figure 2C). (**B**) According to the current genome annotation system, the sequences of 10 *APP* isoforms were selected for demonstration (from top to the bottom) using the Ensembl ID ENST00000354192, -439274, -346798 (APP770-encoding), -348990 (APP695-encoding), -357903 (APP751-encoding), -358918 (APP752-encoding), -359726, -707132, -440126 and -448850. Nine *APP* isoforms can be classified into Classes 1 and 2 (indicated by I and II), while the last isoform (ENST00000448850) is annotated as being transcribed from a transcription initiation site downstream of APP/I58417. Class 1 *APP* isoforms encode signal peptides (indicated by blue *) in their first exons. APP/36594N is ambiguously labeled as splice junction 2, 3, or 4 across different isoforms. The exons and introns of the 10 isoforms are listed in Table S5.

Known genomic features—such as exons, isoforms, and genes—refer to those annotated in genome databases, particularly those assigned Ensembl IDs. To support the new method, DSFA, we designed the Nankai naming system for naming genomic features. The Nankai naming system assigns names to genomic features based on their length using the format Gene/Ex for exons, Gene/Iy for introns and Gene/yN for splice junctions, where x and y represent their respective lengths in base pair (bp). The new naming system guarantees unique identifiers with only extremely rare overlaps. With this new system, 20 known exons, 24 introns, and 24 splice junctions of the *APP* gene can be displayed in a single, simplified figure (Figure 1A). Among them, APP/I58417 denotes a unique intron of the gene *APP* that spans 58,417 bp in the human genome and encodes the APP protein—the precursor of amyloid-beta (Aβ). Correspondingly, APP/58417N denotes a unique splice junction, where the two flanking exons of APP/I58417 are joined in the mature RNA, with APP/I58417 having been excised. APP/36594N is another splice junction that is ambiguously labeled as splice junction 2, 3, or 4 across different isoforms in the current annotation system (Figure 1B). Comparing Figure 1A,B illustrates the similarities and differences between the new naming system and the current genome annotation system.

Splice junctions can be identified by counting splice-junction (sj) reads that align properly to these junctions, adhering to a set of criteria (Figure S1) designed to minimize false positives. Thus, a matrix composed of sj read counts (termed as JRC matrix) can be derived from an RNA-seq dataset to represent the occurrences of splice junctions in the examined samples. In this matrix, the columns (i.e., samples) list the number of splicing events at every junction (or a selected set), yielding a splicing-occurrence profile/signature, while rows (i.e., splice junctions) show their occurrences across all samples. In a normalized JRC matrix (Section 2), the scaled expression level of a splice junction can be interpreted as the relative frequency of splicing events that have occurred at this splice junction, hereinafter referred to as junction frequency, or simply, usage. DSFA then scans these frequencies to flag splice junctions whose usage differs significantly between conditions, i.e., DE splice junctions (Table 1). DSFA is primarily applied to non-DE genes, which are often overlooked in traditional DGEA-based workflows. DSFA compares junction frequencies within each non-DE gene to identify DE splice junctions. Although DSFA may also be applied to DE genes, this remains an open question. Moreover, the non-DE/DE boundary is inherently blurred: it hinges on the chosen statistical model, where dispersion estimates or significance cut-offs are applied. Consequently, a gene identified as DE in one dataset may be identified as non-DE in another, even when the same biological contrast is examined.

According to human genome annotations (Section 2), all *APP* isoforms can be classified into two classes, designated as Classes 1 and 2 (Figure 1B), which encode secretory and non-secretory proteins, respectively. Secretory APP proteins contain a 17-amino acid (aa) signal peptide MLPGLALLLLAAWTARA that mediates protein secretion. APP770, APP751, and APP695—the three major APP proteins that have been the exclusive focus of almost all APP-directed studies—represent typical examples of Class 1. Non-secretory APP proteins lack this signal peptide [15] and are typically encoded by isoforms containing rare splice junctions that occur at extremely low frequencies. Based on this classification criterion, Class 1 isoforms may contain APP/80491N or APP/58417N as their first splice junctions, which are formed through the excision of APP/I80491 and APP/I58417—the largest and second-largest introns, respectively, among all introns in the six AD-associated genes—while Class 2 isoforms may contain APP/58838N, APP/41847N, or APP/28006N as their first splice junctions (Figure 1B). Among these rare junctions, the upstream first exon of APP/28006N may encode a non-signal peptide MDQLEDLLVLFINY, while those of APP/58838N and APP/41847N do not encode any peptides. In contrast to humans, only App/52804N (orthologous to human APP/58417N) has been identified as the first splice junction in mouse Class 1 *APP* isoforms. The *APP* gene’s hallmark—a short first exon encoding a signal peptide followed by an exceptionally long first intron (Figure 1A)—is highly conserved among animals, despite considerable divergence in the aa sequences of the signal peptides. A notable example is the signal peptide of APP (RefSeq: NM_057278) in *Drosophila melanogaster* (fruit fly) MCAALRRNLLLRSLWVVLAIGTAQVQ|A. Similar signal peptides of APP or APP-like proteins have been identified in mouse (RefSeq: NM_001198823), rat (RefSeq: NM_019288), zebrafish (RefSeq: NM_131564), and worm (RefSeq: NM_076470). Functionally, Class 1 isoforms containing human APP/58417N or mouse App/52804N are predominantly responsible for translating secretory APP proteins; therefore, the usage of these junctions reflects the relative proportion of secretory *APP* isoforms.

### 3.2. Large-Scale RNA-Seq Data Mining Using DSFA

Upon re-examining the previous results derived from public RNA-seq datasets (Section 2), we found no consistency in up- or down-regulated DE genes reported by these DGEA-based studies—a finding confirmed by our own re-analysis using DGEA. Notably, the six AD-associated genes (*APP*, *MAPT*, *PSEN1*, *PSEN2*, *APOE*, and *CLU*) were repeatedly identified as non-DE genes. Then, we re-analyzed these RNA-seq datasets (Table S1) using DSFA, focusing on the six AD-associated genes. Overall, our re-analysis uncovered several minor findings that were inconsistent with those reported in the previous study on tau pathology [9]. Specifically, *APP* exhibited the largest number of splice junctions (Table 1) and harbored nearly all of the identified novel and DE splice junctions among the six AD-associated genes. In contrast, the previous study [9] reported that *APOE*, rather than *APP*, exhibited the largest number of isoform types. Furthermore, although both previous [9] and present studies identified a substantial number of novel splice junctions within the AD-associated genes, nearly all were supported by very few sj reads. These rare splice junctions require further verification using full-length transcriptome sequencing.

**Figure 2:**
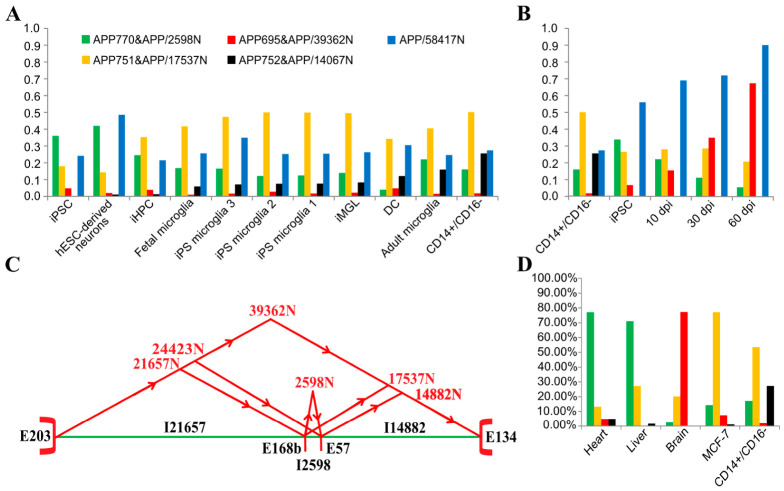
Tissue- and cell-specific expression pattern of *APP* isoforms. Four major *APP* isoforms (APP770-, APP751-, APP695-, and APP752-encoding) and the APP714-encoding isoform can be represented by APP/2598N, APP/17537N, APP/39362N, APP/14067N, and APP/24423N, respectively. (**A**) the usage (i.e., relative frequency) of splice junctions representing five *APP* isoforms was calculated using RNA-seq data (SRA: SRP092075). (**B**) the usage of splice junctions representing five *APP* isoforms was calculated using RNA-seq data (SRA: SRP490059). (**C**) The APP695-, APP751- and APP770-encoding isoforms contain one (i.e., APP/39362N), two (i.e., APP/21657N and APP/17537N) and three splice junctions (i.e., APP/21657N, APP/2598N and APP/14882N), respectively, while the rare APP714-encoding isoform contains APP/24423N and APP/14882N. Here, APP/2598N denotes the splice junction 2598N of the *APP* gene. (**D**) The relative proportions of four major *APP* isoforms in human brain, heart, and liver tissues and MCF-7 cells were calculated using PacBio cDNA-seq data, while that in CD14+/CD16− cells was calculated by transformation of results derived from RNA-seq data (SRA: SRP092075). iPSC: induced pluripotent stem cell; hESC: human embryonic stem cell; iHPC: hematopoietic progenitor from differentiated from iPSC; iMGL: microglial-like cell differentiated from iPSC; DC: dendritic cell; CD14+/CD16−: CD14 positive and CD16 negative cell, i.e., monocytes; iPS microglia 1, 2, and 3: iMGL co-culture without or with rat hippocampal neurons, and TGFB withdrawal.

Applying DSFA to public RNA-seq datasets, we identified human APP/58417N and its mouse ortholog App/52804N as DE splice junctions. Across a considerable fraction of the re-analyzed RNA-seq datasets, we consistently observed decreased usage of these junctions in AD samples from neurons, microglia and hippocampal tissues (Table 2), which implies reduced inclusion of the first exon encoding a signal peptide (Figure 1B). Conversely, we observed increased usage of human APP/14067N and its mouse ortholog App/12293N in AD samples. The changes did not reach statistical significance, most likely due to increased variance arising from low baseline APP/14067N and App/12293N usage values. Furthermore, APP/14067N and App/12293N are highly expressed in immune cells, particularly monocytes (Figure 2A), relative to other cell types. Specifically, in an RNA-seq dataset (SRA: SRP092075), the average relative frequency of APP/14067N in monocytes is 1.6-fold higher than that in adult microglia, which in turn exceeds that in hESC-derived neurons and iPSCs by more than 20-fold and 117-fold, respectively. As APP/14067N corresponds to APP752-encoding isoforms, APP752 exhibits immune cell specificity—this represents the first important finding of the present study.

Based on the first important finding, APP/2598N can represent the APP770-encoding isoform in non-immune cells, as the APP752-encoding isoform also containing APP/2598N is expressed at extremely low levels. The APP695- and APP751-encoding isoforms can be represented by two specific splice junctions, APP/39362N and APP/17537N, respectively (Figure 2C). As revealed by re-analyzing an RNA-seq dataset (SRA: SRP490059), the relative proportion of the APP695-encoding isoform increased, whereas that of the APP770-encoding isoform decreased, during the differentiation of iPSCs into neurons (Figure 2B), resulting in an increased proportion of APP695 among all *APP* isoforms. Similarly, the relative proportion of the APP752-encoding isoform increased during the differentiation of iPSC into microglia (Figure 2A), akin to the increase in the relative proportion of the APP695-encoding isoform during its differentiation. Additionally, the relative proportion of the APP751-encoding isoform decreased significantly only in iPSC-derived neurons at 60 days post-induction (dpi). The APP/58417N usage also increased during the differentiation of iPSCs into neurons, which was accompanied by an increase in the overall expression of the *APP* gene. Such a dual increase in *APP* expression and the APP/58417N usage ensures sufficient production of secretory APP proteins, which may be required for neuronal differentiation. Additionally, APP/58417N usage in neurons is substantially higher than that in other cell types (Figure 2A,B). Unexpectedly, APP/39362N and APP/2598N usage significantly increased and decreased, respectively, in iPSC-derived neurons at 60 dpi from AD patients compared to their age-matched controls, while APP/58417N usage did not significantly differ. The somatic origins of these iPSCs were dermal fibroblasts from late-onset sporadic AD patients [16]. However, when the somatic origins were dermal fibroblasts from patients with familial AD (FAD)-associated mutations APP/V717I, PS1/A79V, and PS2/N141I [17], the APP/58417N usage in iPSC-derived neurons decreased, compared to the controls (SRA: SRP382946).

### 3.3. Differential Expression of APP Isoforms and Splice Junctions

**Figure 3:**
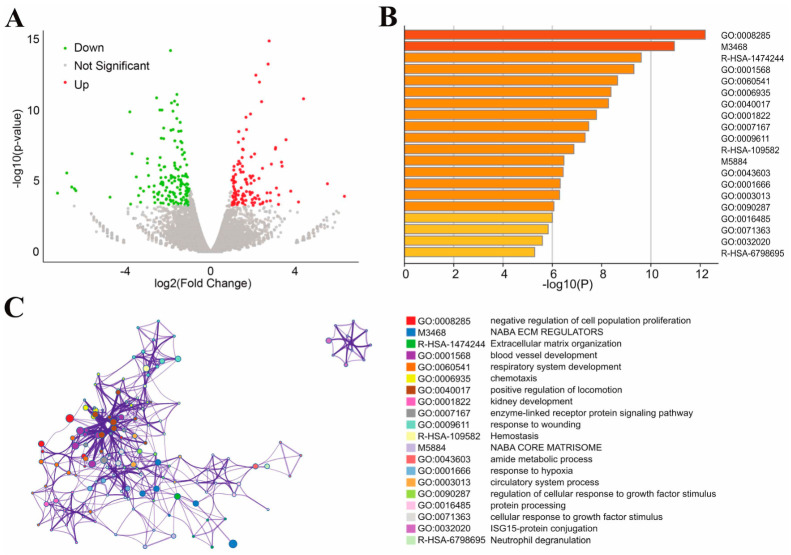
U1 snRNA over-expression in human hESC-derived neurons. Two groups of the human embryonic stem cell (hESC)-derived neurons were used in the experiments (Section 2): the U1 group over-expressing U1 snRNA, and the control group (SRA: SRP178463). (**A**) Using differential gene expression analysis (DGEA), 48 significantly up-regulated and 52 down-regulated genes (100 in total) were identified between the U1 and control groups (Table S4). (**B**) After removal of lncRNAs, all DE protein-coding genes were functionally annotated and analyzed using https://metascape.org/. These genes were enriched in 20 entries from Gene Ontology, MsigDB, and Reactome databases. (**C**) Two networks were constructed using the 20 entries.

Encouraged by our earlier observation that U1 snRNA over-expression results in highly similar AD-like phenotypes (increased Aβ_42/40_ ratio and pTau212, as well as impairments of synaptic plasticity and spatial memory) [14] by influencing RNA splicing, we hypothesized that U1 snRNA over-expression decreases APP/58417N usage. To test this hypothesis, we over-expressed U1 snRNA in hESC-derived neurons and obtained a new RNA-seq dataset (SRA: SRP178463) for the present study. Comparison of U1-snRNA-over-expressing and control groups using DGEA (Section 2) identified a set of 48 up-regulated and 52 down-regulated genes or lncRNAs from a total of 25,283 protein-coding genes and lncRNAs (Table S4). Within the 100 genes or lncRNAs in this DE gene set (Figure 3A), 33 participate in negative regulation of cell population proliferation (GO: 0008285) (Figure 3B). Among the 33 genes or lncRNAs, *NOTCH3*, *ADAM19*, *ID1*, and *ID3* are involved in the Notch signaling pathway, while *NOTCH1* and *HES1* were also up-regulated, though not significantly. Network analysis reveals that GO: 008,285 resides at a relatively isolated node (Figure 3C). However, none of the six AD-associated genes were present in the DE gene set. We therefore performed DSFA on the six AD-associated genes using the U1-snRNA-over-expressing dataset. As a result (Table 1), U1 snRNA over-expression significantly decreased the APP/58417N usage in neurons to 85.26% (logFC = −0.23, *p* < 0.01), similar to the effects observed in AD samples. RT-qPCR (Section 2) confirmed this decrease to 82.9% (logFC = −0.27, *p* < 0.01). Moreover, U1 snRNA over-expression increased APP/14067N and APP/39362N usage in neurons. These changes did not reach statistical significance, requiring further investigation given the low sequencing depth coverage of these rare splice junctions.

As an additional finding, U1 snRNA over-expression increased the usage of rare splice junctions. The increased usage of APP/39362N and APP/17537N relative to APP/2598N is such an example (Figure 1A). This shift favors the generation of isoforms encoding APP695 and APP751 relative to APP770, offering a possible explanation for the subtle increase in APP751 reported in the AD frontal cortex in the previous study [18], which compared expression levels and alternative splicing of *APP* isoforms encoding APP770, APP751, and APP695 in brain tissue from hAPP transgenic and nontransgenic mice and from humans with and without AD. In the U1-snRNA-over-expressing RNA-seq dataset, only three novel rare splice junctions, APP/56921N, APP/5198N, and APP/69N (Table 1), barely exceeded the coverage cutoff and were included in the statistical analysis. In contrast, the remaining 90 rare splice junctions were excluded due to average coverage of less than one sj read per sample. The corresponding rare introns APP/I56921, APP/I5198, and APP/I69 are located within the relatively large and high-frequency introns APP/I58417 and APP/I9950, as well as the exon APP/E203, respectively. Alternative splicing of APP/I9950 generates APP/I4673, APP/E79, and APP/I5198, while that of APP/E203 generates APP/E46, APP/I69, and APP/E88 (Figure S2). Additionally, alternative splicing of APP/I58417 generates APP/I56921 and a novel upstream exon, suggesting an alternative 5′ splice site or transcription initiation site (TIS). Although the 5′ end of this site has not been determined, a non-signal peptide MLRSCLHDSWARGGCISLRTS could be translated from the 5′ end of the coding region in this new isoform, indicating that it encodes a non-secretory APP protein.

Decreased usage of APP/58417N and App/52804N is likely attributable to the increased formation of Class 2 *APP* isoforms relative to Class 1. According to human genome annotations, Class 2 isoforms include those containing alternative 5′ splice sites or TISs, such as isoforms containing APP/58838N, APP/41847N, or APP/28006N, as well as short *APP* isoforms (e.g., Ensembl: ENST00000448850) transcribed from TISs downstream of APP/I58417 (Figure 1B). Another possible driver is novel isoforms, which may be generated by alternative TISs within or downstream of APP/I58417 and App/I52804, premature termination of transcription, or splicing defects between their adjacent exons. However, we did not detect expression of known Class 2 *APP* isoforms or novel splice junctions with expression levels higher than those of APP/80491N or APP/28006N using multiple transcriptomic data such as PARE-seq (SRA: SRP007508), PA-seq (SRA: SRP017395), and GRO-seq (SRA: SRP095085). In addition, we detected a few potential novel TISs within APP/I58417 using CAGE-seq data (SRA: DRP000372). However, any single one of the detected Class 2 or novel isoforms is insufficient to account for such a significant decrease in APP/I58417 or App/I52804 usage due to its exceedingly low expression level. The last possible driver is premature cleavage and polyadenylation (PCPA) [19], but we failed to detect any PCPA sites, seemingly due to the limited sensitivity of short-read methods for detecting rare 3′-end formation events. To overcome this limitation, we turned to public PacBio cDNA-seq (e.g., SRA: SRP067402) and HIDE-seq data, However, none of these high-resolution resources provided unambiguous evidence for PCPA or alternative termination within APP/I58417. Besides encoding a signal peptide, these first exons (e.g., APP/E207 for human) are also characterized by exceptionally high enrichment of CGG repeats compared to other regions of the *APP* gene. Expanded CGG repeats have been linked to neurodevelopmental and neurodegenerative disorders [20]. However, no ac4C modification was detected on these CGG repeats by re-analysis of a RIP-seq dataset (SRA: SRP544711), despite their alignment with the CXX motif.

Using public PacBio cDNA-seq data from human brain, heart, and liver tissues and MCF-7 cells (Figure 2D), we detected only the APP770-, APP751-, APP695-, APP752-, and APP714-encoding isoforms (Supplementary File S3). Other annotated isoforms—particularly those containing rare splice junctions (e.g., APP/80491N, APP/28006N, and APP/8614N)—were not detected. Among the five detected isoforms, the APP714-encoding isoform (Figure 2C), which has not been included in any database or assigned an Ensembl ID, was detected only in brain tissue. Consistent with prior knowledge, the APP695-encoding isoform predominates in neural tissues (e.g., brain), while the APP770-encoding isoform predominates in non-neural tissues (e.g., heart and liver). An important finding of the present study (Figure 2D) is that the APP751-encoding isoform predominates in both cancer (e.g., MCF-7) and immune cells (e.g., monocytes). Other findings include: (1) the APP752-encoding isoform ranks as the fourth most abundant, following the three major isoforms (APP770-, APP751-, and APP695-encoding); (2) the APP752-encoding isoform is the second most abundant in immune cells; (3) two TTSs, designated TTS1 and TTS2, were identified within *APP*, giving rise to two distinct alternative last exons (E1222 and E996), respectively; and (4) although a novel transcript terminating with AAAAAAAAAAAAAAAAAAAAAGA was detected within intron APP/I21657, its termination site is unlikely to represent a true TTS. TTS1 is the only annotated TTS of *APP*, whereas TTS2, which features a polyA-like site ACATAAATAAATTAAATAAAATAA at its 3′ end, is located upstream of TTS1. PolyA-like sites similar to TTS2 that function as TTSs were reported in our previous studies of mitochondrial [21] and chloroplast genomes [22]. Notably, the APP752-encoding isoform is terminated at TTS2 in MCF-7 cells but at TTS1 in heart and liver tissues.

### 3.4. Expanded Analysis of APP/58417N and App/52804N Usage

**Figure 4:**
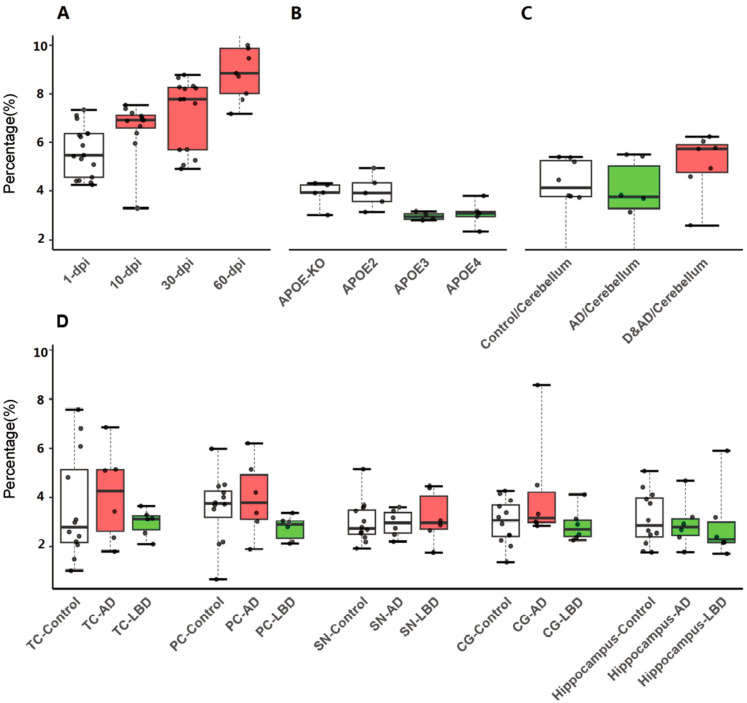
The usage changes of APP/58417N and App/52804N in various tissue or cell types. The increased and decreased changes are indicated by red and green boxes, respectively. Blank boxes represent control or no change. (**A**) Each box contains data from the induced pluripotent stem cell (iPSC) lines from AD patient-derived and their age-matched controls. (**B**) Human microglial with *APOE* knockout (APOE-KO) were isolated from xenotransplanted AD mouse models and used as controls. (**C**) Cerebellum was obtained from AD, D&AD patients or their controls. (**D**) These tissues were obtained from AD, LBD patients or their controls. dpi: day post-induction; APOE2-4: human microglial expressing the type 2-4 *APOE* gene; AD: Alzheimer’s disease; D&AD: Down syndrome with Alzheimer’s disease; LBD: Lewy body dementia; TC: temporal cortex; PC: parietal cortex; SN: substantia nigra; CG: cingulate gyrus.

Although the decreased expression of secretory *APP* isoforms was not confirmed by PacBio cDNA-seq data analysis, it still merits further research for the following reasons: (1) a correlation showing decreased usage of APP/58417N and App/52804N in some RNA-seq datasets, especially those generated from cellular samples carrying FAD-associated mutations; and (2) a causality demonstrating that U1 snRNA over-expression directly reduces APP/58417N usage. These two points can be bridged by our previous finding that some FAD-associated mutations (e.g., PS1∆E9) increase U1 snRNA expression, which is associated with AD [14]. Additionally, the increased U1 snRNA expression detected in AD patients is well-documented [23,24]. Together, these findings are consistent with an expanded analysis of public RNA-seq data (Table 2). For example, the APP/58417N usage in the iPSC-derived neurons decreased, compared to the controls (SRA: SRP382946), when the somatic origin of the iPSCs was dermal fibroblasts from patients with FAD-associated mutations APP/V717I, PS1/A79V, and PS2/N141I. In contrast, the APP/58417N usage did not significantly differ between AD patient-derived iPSC lines and their age-matched controls at 1, 10, 30, or 60 dpi (Figure 4A), as revealed by re-analyzing another RNA-seq dataset (SRA: SRP490059). This unexpected result likely stemmed from the somatic origin of the iPSCs—dermal fibroblasts from late-onset sporadic AD patients. Beyond *APP*, whether APP/58417N usage is modulated by mutations in other AD-associated genes remains unexplored, simply because of the lack of public data. Across the few existing datasets, DSFA was used for other genes as it had been for *APP*. For example, the effects of the human APOE isoforms (*APOE2*, *APOE3*, and *APOE4*) and the *APOE* knockout (APOE-KO) on the microglial response to Aβ pathology in brains of AppNL-G-F mice were investigated using both RNA-seq and ATAC-seq (SRA: SRP517721). Re-analysis of the RNA-seq data alone uncovered a significant decrease in APP/58417N usage in both the *APOE3* and *APOE4* groups compared to the APOE-KO group (Figure 4B). In contrast, no such decrease was observed in the *APOE2* group. Additional datasets are required to validate this APOE-driven effect.

The above cell-level findings clarify the paradoxical shifts or lack of change in APP/58417N and App/52804N usage in tissue samples that we originally detected in our large-scale RNA-seq data mining (Table 2). For example, by re-analyzing an RNA-seq dataset (SRA: SRP447810), we detected a significant decrease in the App/52804N usage in the hippocampus from 5-month 5xFAD mice but not in the hippocampus from 3-, 6- and 7-month-old 5xFAD mice from other RNA-seq datasets (SRA: SRP543852, SRP555827 and SRP521327), respectively. This inconsistency reflects two intrinsic limitations of tissue-level comparisons: (1) the average *APP* expression differs up to more than 37-fold across oligodendrocytes, endothelial cells, neurons, pericytes, and nine other cell types [25], and therefore, tissue-level averaging masks any cell-level change; and (2) the five FAD-associated mutations carried by 5xFAD mice influence U1 snRNA expression in opposite directions. By re-analyzing another RNA-seq dataset (SRA: ERP161086), we detected a decrease in the APP/58417N usage in the cerebellum of AD patients and an increase in the cerebellum of AD patients who were also diagnosed with Down syndrome (Figure 4C). However, these changes were not observed in the prefrontal cortex of either group. Another study (SRA: SRP540706) was conducted to examine the changes in the APP/58417N usage across various brain regions, including the temporal cortex (TC), parietal cortex (PC), substantia nigra (SN), cingulate gyrus (CG), and hippocampus (Table 2). As expected, we detected a decrease in the APP/58417N usage in the hippocampus of AD patients compared to their respective controls. However, the changes in the APP/58417N usage exhibited large regional heterogeneity across the brain. Specifically, the APP/58417N usage was lowest in hippocampus and SN and rose in the PC, TC, and CG of the AD patients, while it was lowest in the PC and rose in the CG, hippocampus, TC, and SN of the Lewy body dementia (LBD) patients (Figure 4D). On average, the APP/58417N usage was significantly lower in the brain regions of LBD patients than in those of AD patients. The above findings showed that tissue samples are too heterogeneous to probe molecular mechanisms as refined as ours. Therefore, additional experiments utilizing specific cell types containing only one mutation are necessary to precisely quantify the contribution of each cell or mutation type.

## 4. Discussion

In the present study, we developed DSFA to detect DE splice junctions using RNA-seq data, potentially obtaining novel findings that were overlooked in previous studies. This strategy provides a new research direction, termed DSFA-driven discovery. However, DSFA has only been applied to six non-DE genes; its utility for DE genes needs to be validated. When DSFA is extended to DE genes, splice junctions identified as non-DE must be interpreted cautiously, as they still contribute to the overall gene-level expression changes. The DE splice junctions identified by DSFA, particularly the rare and novel ones (e.g., APP/56921N, APP/5198N, and APP/69N), require confirmation by PacBio cDNA-seq data analysis. Such confirmation also demonstrated that APP770-, APP751-, APP695-, APP752-, and APP714-encoding isoforms can be represented by APP/2598N, APP/17537N, APP/39362N, APP/14067N, and APP/24423N, respectively. These splice junctions enable greatly simplified characterization of tissue- and cell-specific *APP* isoform expression.

Despite the identification of numerous novel isoforms using Nanopore RNA-seq and novel splice junctions using RNA-seq in both the previous [9] and present studies, only APP770-, APP751-, APP695-, APP752-, and APP714-encoding isoforms were detected using PacBio cDNA-seq data. Although increasing sequencing depth can indeed enable the detection of more rare isoforms, their changes are unlikely to exert significant physiological or pathological effects. The same holds true for the APP714-encoding isoform, which was detected only in brain tissue at extremely low levels. Consequently, the APP770-, APP751-, APP695-, and APP752-encoding isoforms were determined as major isoforms. Previous AD research has focused on the APP695-encoding isoform in neurons; however, the APP752-encoding isoform in immune cells has received no attention. Compared with the APP770-encoding isoform, the APP752-encoding isoform lacks the exon E54 (Figure 1A), resulting in the absence of the 18-aa segment EPVDARPAADRGLTTRPG, which is located 16-aa from the Aβ segment. Among the five detected isoforms, the APP752-encoding isoform is the most likely to affect Aβ cleavage due to the absence of this segment very close to the Aβ segment, which merits future investigation. Although neurons are widely accepted as the primary source of Aβ until a new possible origin is proposed [25], the subcellular site of Aβ production is still under debate [26]. Currently, it is widely accepted that APP695 serves as the primary source of Aβ because it predominates in neurons within brain tissue [27]. However, the discovery that the APP752-encoding isoform is the second most abundant in microglia—which, like neurons, are distributed throughout brain tissue—may pose a new challenge.

Although we did not confirm a decreased proportion of secretory APP isoforms or increased expression of non-secretory isoforms using PacBio full-length transcriptome data, this represents only one predicted cause for the decreased usage of APP/58417N and App/52804N. Other potential causes remain to be investigated. For example, because APP/58417N and App/52804N are located near the 5′ ends of mRNAs, differential degradation proportions across samples can affect analysis of their differential usage—a limitation inherent in the library construction process of all transcriptome sequencing technologies. Furthermore, whether U1 snRNA over-expression causes differential degradation of mRNAs is also unknown. Thus, the molecular mechanisms underlying the decreased usage of APP/58417N and App/52804N remain unclear. Nevertheless, the decreased usage of these junctions reflects a decreased proportion of secretory *APP* isoforms, as the exon encoding the signal peptide lies upstream of both APP/58417N and App/52804N. Confirmation of this finding may help explain the intracellular Aβ accumulation previously observed in neuronal cells, an early event in AD pathogenesis [28], providing insights into the long-standing debate over its origin—whether from intracellular APP proteolysis or receptor-mediated uptake of extracellular Aβ.

## 5. Conclusions

For isoform identification, PacBio full-length transcriptome sequencing remains the only reliable technology to date. Despite the identification of numerous novel isoforms by Nanopore RNA-seq or splice junctions by conventional RNA-seq, only a handful of major isoforms per gene are likely to be of genuine biological significance. The application of DSFA to large-scale RNA-seq data mining enables effective detection of differential isoform expression across tissues, cells, and other conditions (e.g., AD), thereby facilitating the discovery of potential biomarkers and therapeutic targets. For the first time, we recognize that the major isoforms of a gene can differ this markedly in their expression across tissue and cell types. Consequently, compared with the well-studied field of tissue-specific gene expression, research on differential isoform expression holds greater value.

## Figures and Tables

**Table 1:** DSFA result of the *APP* gene.

Splice Junctions	logFC	logCPM	*p* Value	FDR
APP/69N *	8.01	8.77	7.34 × 10^−16^	1.98 × 10^−14^
APP/725N	0.03	15.81	4.26 × 10^−1^	5.96 × 10^−1^
APP/734N	0.51	6.92	7.82 × 10^−1^	8.12 × 10^−1^
APP/937N	0.06	15.64	6.96 × 10^−2^	2.35 × 10^−1^
APP/2042N	0.07	15.64	4.65 × 10^−2^	2.35 × 10^−1^
APP/2598N	0.07	15.36	2.15 × 10^−1^	4.46 × 10^−1^
APP/5198N *	6.67	7.64	2.58 × 10^−7^	3.49 × 10^−6^
APP/5704N	−0.03	16.05	3.58 × 10^−1^	5.71 × 10^−1^
APP/6315N	−0.02	15.67	5.15 × 10^−1^	6.33 × 10^−1^
APP/6663N	−0.04	15.60	3.25 × 10^−1^	5.71 × 10^−1^
APP/7350N	−0.03	15.60	4.04 × 10^−1^	5.96 × 10^−1^
APP/8614N	0.49	7.35	6.64 × 10^−1^	7.47 × 10^−1^
APP/9950N	−0.06	16.38	6.27 × 10^−2^	2.35 × 10^−1^
APP/14067N	0.83	10.18	8.05 × 10^−3^	5.43 × 10^−2^
APP/14882N	0.05	15.44	3.60 × 10^−1^	5.71 × 10^−1^
APP/17537N	0.23	13.81	3.12 × 10^−1^	5.71 × 10^−1^
APP/19314N	0.04	16.12	1.80 × 10^−1^	4.42 × 10^−1^
APP/21657N	0.00	16.06	9.27 × 10^−1^	9.27 × 10^−1^
APP/21906N	−0.06	16.16	9.70 × 10^−2^	2.91 × 10^−1^
APP/24423N	−0.94	8.24	6.89 × 10^−2^	2.35 × 10^−1^
APP/28957N	0.04	16.22	2.15 × 10^−1^	4.46 × 10^−1^
APP/36594N	0.02	15.15	7.54 × 10^−1^	8.12 × 10^−1^
APP/39362N	0.20	11.02	4.77 × 10^−1^	6.14 × 10^−1^
APP/42627N	0.02	15.81	4.41 × 10^−1^	5.96 × 10^−1^
APP/56921N	4.11	6.18	1.29 × 10^−1^	3.49 × 10^−1^
APP/58417N *	−0.23	15.57	3.95 × 10^−5^	3.56 × 10^−4^
APP/80491N	−0.73	6.84	5.61 × 10^−1^	6.58 × 10^−1^

Analogous to differential gene expression analysis (DGEA), differential splicing frequency analysis (DSFA) of SRP178463 was performed to identify significantly differentially expressed (DE) splice junctions (red indicates increase and green indicates decrease upon U1 snRNA over-expression). After 90 rare splice junctions were removed, the expression levels of 27 splice junctions were compared between the cell group over-expressing U1 snRNA and the control group. Among these 27 splice junctions, 24 known splice junctions were frequently detected across different samples, while the remaining 3 (indicated by *) were rare splice junctions. Among the three rare splice junctions, APP/69N and APP/5198N were identified as DE splice junctions by DSFA. Among the 24 known splice junctions, only APP/58417N was identified as a DE splice junction. LogFC (Log Fold Change) is the logarithmic (base 2) transformation of the fold change between two conditions or groups; LogCPM (Log Counts Per Million) is the logarithm of the average normalized gene expression values across all samples, where the normalization is done by scaling the raw counts to counts per million (CPM); PValue (*p*-value) and FDR (False Discovery Rate), also referred to as the adjusted *p*-value, were calculated using the edgeR package (Section 2).

**Table 2:** Selected RNA-seq datasets in large-scale data mining.

SRA Acc	Species	Tissue/Cell	Group (Sample Number)	DE Splice Junction
SRP447810	Mouse	Hippocampus	AD (6), W/C (6)	App/52804N
SRP543852	Mouse	Hippocampus	AD (3), W/C (3)	NA
SRP555827	Mouse	Hippocampus	AD (3), W/C (3)	NA
SRP521327	Mouse	Hippocampus	AD (3), W/C (3)	NA
SRP490059	Human	iPSC-derived neurons	AD (28), W/C (24)	APP/58417N
SRP382946	Human	iPSC-derived neurons	AD (3),W/C (9)	APP/58417N
SRP517721	Human	Microglia	APOE-KO (5), APOE2 (5), APOE3 (4), APOE4 (5)	APP/58417N
ERP161086	Human	Cerebellum and prefrontal cortex	AD (13), D&AD (14), W/C (15)	APP/58417N *
SRP540706	Human	Hippocampus, SN, CG, PC, and TC	AD (30), W/C (60), LBD (30)	APP/58417N *
SRP178463	Human	hESC-derived neurons	U1 over-expressing (2), W/C (2)	APP/58417N

The 10 RNA-seq datasets presented in this table are representative of a total of 118 human and 187 mouse bulk RNA-seq datasets associated with Alzheimer’s disease (AD). One of the ten RNA-seq datasets (SRA: SRP178463) was generated in the present study and nine other datasets were generated from previous studies. Detailed information regarding the remaining datasets and the corresponding results are provided in Supplementary Files S1 and S2, respectively. * The changes in the usage of APP/58417N are complex, involving both decreases and increases. SRA Acc: accession number in the NCBI SRA database; DE splice junction: differentially expressed splice junctions between AD and control or wild-type samples; iPSC: induced pluripotent stem cell; hESC: human embryonic stem cell; SN: subtantia nigra; CG: cingulate gyrus; PC: parietal cortex; TC: temporal cortex; W/C: wild type for variants or controls for patients; D&AD: Down syndrome with Alzheimer’s disease; LBD: Lewy body dementia; APOE-KO: *APOE* knockout; APOE2-4: cells expressing the type 2-4 *APOE* gene; U1 over-expressed: cells over-expressing U1 snRNA.

## Data Availability

The raw reads of four samples are openly available in the NCBI SRA database under the project accession number SRP178463. The gene expression matrix of SRP178463, generated according to Ensembl annotations release 114, is available in the NCBI GEO database under the series accession number GSE124951. All other data used in the present study were obtained from public databases, and the identifiers (IDs) for all these data are provided in the Supplementary Files.

## Supplementary Materials

The following supporting information can be downloaded at: https://www.mdpi.com/article/10.3390/cells15121086/s1, Supplementary File S1: Figures S1 and S2 and Tables S1–S5; Supplementary File S2: Selected results of large-scale RNA-seq data mining; Supplementary File S3: Selected results of PacBio cDNA-seq data analysis.
