# Supplementary material for: Investigating Alzheimer’s Disease-Associated Genes Using Differential Splicing Frequency Analysis
Source: Cells. 2026 Jun 15;15(12):1086. doi: 10.3390/cells15121086 (PMC13297122; doi:10.3390/cells15121086)
Supplement: Supplementary file 1 [file cells-15-01086-s001.zip › S1.pdf]

# Supplementary S1

**Table S1. RNA-seq datasets for this study**

| Human | Project Acc | Raw data Acc | Description |
|---|---|---|---|
| 1 | PRJNA1099279 | NA | |
| 2 | PRJNA1214194 | SRP559003 | Small data size |
| 3 | PRJNA851439 | SRP382946 | Analyzed |
| 4 | PRJNA1066402 | SRP484589 | Small data size |
| 5 | PRJNA801614 | SRP357209 | Only two samples |
| 6 | PRJNA1004304 | SRP454531 | Only four samples |
| 7 | PRJNA1168520 | SRP536444 | Analyzed |
| 8 | PRJNA1171307 | SRP537690 | Only four samples |
| 9 | PRJNA1001822 | SRP453120 | Low quality data |
| 10 | PRJNA1177488 | SRP540706 | Analyzed |
| 11 | PRJNA1022484 | SRP463932 | scRNA-seq |
| 12 | PRJNA1022482 | SRP463940 | Small data size |
| 13 | PRJNA1022480 | SRP463938 | Small data size |
| 14 | PRJNA975472 | SRP439197 | snRNA-seq |
| 15 | PRJNA1099636 | SRP501458 | Analyzed |
| 16 | PRJNA1126662 | SRP515320 | RNA-seq+ATAC-seq |
| 17 | RRJNA1077346 | SRP490059 | Analyzed |
| 18 | PRJNA1131282 | SRP517721 | Analyzed |
| 19 | PRJNA1131283 | SRP517720 | ATAC-seq |
| 20 | PRJNA1126680 | SRP515319 | Cancer data |
| 21 | PRJNA1111843 | SRP507928 | Analyzed |
| 22 | PRJNA990747 | SRP447154 | Small data size |
| 23 | PRJNA1031180 | SRP468045 | Only four samples |
| 24 | PRJNA967216 | SRP435888 | Small data size |
| 25 | PRJNA967217 | SRP435904 | scRNA-seq |
| 26 | PRJNA974344 | SRP438579 | Analyzed |
| 27 | PRJNA974166 | SRP438538 | Analyzed |
| 28 | PRJNA1023967 | SRP464538 | Only two samples |
| 29 | PRJNA1076936 | SRP489930 | Analyzed |
| 30 | PRJNA857283 | SRP385693 | Small data size |
| 31 | PRJNA1010350 | SRP457349 | Small data size |
| 32 | PRJNA1071604 | SRP487212 | Small data size |
| 33 | PRJNA1069012 | SRP485933 | scRNA-seq+ATAC-seq+RNA-seq |
| 34 | PRJNA1068082 | SRP485432 | Analyzed |
| 35 | PRJNA1067726 | SRP485300 | Analyzed |
| 36 | PRJNA1016483 | SRP460362 | Analyzed |
| 37 | PRJNA1057837 | SRP480332 | Analyzed |
| 38 | PRJNA394839 | SRP112708 | Analyzed |

| | | | |
|---|---|---|---|
| 39 | PRJNA1033674 | SRP469207 | Single-end data |
| 40 | PRJNA964438 | SRP435274 | Analyzed |
| 41 | PRJNA944178 | SRP427033 | Small data size |
| 42 | PRJNA881750 | SRP397912 | RNA-seq+ATAC-seq |
| 43 | PRJNA849203 | SRP380203 | Small data size |
| 44 | PRJNA856464 | SRP385236 | Only two samples |
| 45 | PRJNA851057 | SRP382717 | Analyzed |
| 46 | PRJNA949954 | SRP429938 | Analyzed |
| 47 | PRJNA988027 | SRP446167 | Analyzed |
| 48 | PRJNA876264 | SRP395415 | scRNA-seq+ATAC-seq |
| 49 | PRJNA940831 | SRP425558 | Analyzed |
| 50 | PRJNA925863 | SRP418446 | Small data size |
| 51 | PRJNA925533 | SRP418305 | Cancer data |
| 52 | PRJNA948405 | SRP429133 | Analyzed |
| 53 | PRJNA940797 | SRP425594 | snRNA-seq |
| 54 | PRJNA853055 | SRP383629 | Small data size |
| 55 | PRJNA873113 | SRP393893 | Only four samples |
| 56 | PRJNA912653 | SRP413248 | scRNA-seq |
| 57 | PRJNA920518 | SRP416331 | Small data size |
| 58 | PRJNA918359 | SRP415735 | Analyzed |
| 59 | PRJNA917304 | SRP415453 | snRNA-seq |
| 60 | PRJNA785585 | SRP348927 | snRNA-seq |
| 61 | PRJNA896178 | SRP405487 | Small data size |
| 62 | PRJNA846069 | SRP378528 | Small data size |
| 63 | PRJNA819029 | SRP365478 | scRNA-seq |
| 64 | PRJNA837954 | SRP375143 | Analyzed |
| 65 | PRJNA782006 | SRP346962 | scRNA-seq |
| 66 | PRJNA782007 | SRP346958 | Analyzed |
| 67 | PRJNA855866 | SRP384909 | Analyzed |
| 68 | PRJNA778199 | SRP344764 | scRNA-seq |
| 69 | PRJNA780522 | SRP346150 | Analyzed |
| 70 | PRJNA377888 | SRP101349 | Small data size |
| 71 | PRJNA704612 | SRP307934 | Small data size |
| 72 | PRJNA727602 | SRP318632 | Low quality data |
| 73 | PRJNA773252 | SRP342515 | Disorganized samples |
| 74 | PRJNA767074 | SRP339150 | Analyzed |
| 75 | PRJNA757727 | SRP334089 | Small data size |
| 76 | PRJNA687763 | SRP299450 | Small data size |
| 77 | PRJNA685110 | SRP297875 | Small data size |
| 78 | PRJNA755724 | SRP332931 | Small data size |
| 79 | PRJNA750801 | SRP330498 | Analyzed |
| 80 | PRJNA688060 | SRP299604 | Small data size |
| 81 | PRJNA729525 | SRP319543 | scRNA-seq+ATAC-seq+RNA-seq |
| 82 | PRJNA694049 | SRP302849 | Small data size |

| | | | |
|---|---|---|---|
| 83 | PRJNA650315 | SRP275597 | Small data size |
| 84 | PRJNA689841 | SRP300532 | Only two samples |
| 85 | PRJNA675864 | SRP292025 | Large variation in data size |
| 86 | PRJNA662306 | SRP281197 | Analyzed |
| 87 | PRJNA602538 | SRP243780 | Small data size |
| 88 | PRJNA670209 | SRP287843 | Small data size |
| 89 | PRJNA644127 | SRP270017 | snRNA-seq |
| 90 | PRJNA665286 | SRP285156 | Analyzed |
| 91 | PRJNA662330 | SRP281230 | Analyzed |
| 92 | PRJNA643561 | SRP269599 | Analyzed |
| 93 | PRJNA644383 | SRP270338 | Small data size |
| 94 | PRJNA556201 | SRP216247 | Small data size |
| 95 | PRJNA556200 | SRP216250 | Small data size |
| 96 | PRJNA451437 | SRP142295 | Small data size |
| 97 | PRJNA577618 | SRP225693 | scRNA-seq |
| 98 | PRJNA559812 | SRP218087 | Small data size |
| 99 | PRJNA530241 | SRP190040 | Cancer data |
| 100 | PRJNA527202 | SRP188499 | Analyzed |
| 101 | PRJNA517735 | SRP182857 | Small data size |
| 102 | PRJNA434441 | SRP132992 | Small data size |
| 103 | PRJNA482601 | SRP154995 | Analyzed |
| 104 | PRJNA489467 | SRP159666 | Small data size |
| 105 | PRJNA394980 | SRP112852 | Analyzed |
| 106 | PRJNA484721 | SRP156444 | Analyzed |
| 107 | PRJNA399530 | SRP115959 | Small data size |
| 108 | PRJNA413568 | SRP119561 | Small data size |
| 109 | PRJNA393355 | SRP111293 | Analyzed |
| 110 | PRJNA354375 | SRP093671 | Small data size |
| 111 | PRJNA350562 | SRP092075 | Analyzed |
| 112 | PRJNA339538 | SRP082406 | Analyzed |
| 113 | PRJNA295049 | SRP063449 | Small data size |
| 114 | PRJNA268092 | SRP050084 | Small data size |
| 115 | PRJNA30709 | SRP013565 | Too many samples |
| 116 | PRJNA883236 | SRP398685 | Analyzed |
| 117 | PRJEB76583 | ERP161086 | Analyzed |
| 118 | PRJNA514447 | SRP178463 | Analyzed |

| **Mouse** | **Project Acc** | **Raw data Acc** | **Description** |
|---|---|---|---|
| 1 | PRJNA1090077 | SRP496697 | Only two samples |
| 2 | PRJNA1124066 | SRP513987 | Analyzed |
| 3 | PRJNA1207525 | SRP555827 | Analyzed |

| | | | |
|---|---|---|---|
| 4 | PRJNA1206722 | SRP555373 | Only four samples |
| 5 | PRJNA1205664 | SRP554904 | scRNA-seq |
| 6 | PRJNA1205378 | SRP554800 | RIP-seq |
| 7 | PRJNA1098742 | SRP500826 | Small data size |
| 8 | PRJNA1185211 | SRP544711 | RIP-seq |
| 9 | PRJNA1183411 | SRP543852 | Analyzed |
| 10 | PRJNA1181754 | SRP542957 | Only five samples |
| 11 | PRJNA1127529 | SRP515840 | Small data size |
| 12 | PRJNA1138661 | SRP521329 | Analyzed |
| 13 | PRJNA1138662 | SRP521327 | circRNA-seq+RNA-seq |
| 14 | PRJNA992028 | SRP447810 | circRNA-seq+RNA-seq |
| 15 | PRJNA1163689 | SRP533996 | Analyzed |
| 16 | PRJNA975472 | SRP439197 | snRNA-seq |
| 17 | PRJNA1101554 | SRP502447 | Small data size |
| 18 | PRJNA1035064 | SRP469916 | Only three samples |
| 19 | PRJNA1120790 | SRP512197 | Small data size |
| 20 | PRJNA1118825 | SRP511105 | Analyzed |
| 21 | PRJNA926897 | SRP418815 | Small data size |
| 22 | PRJNA942287 | SRP426302 | Small data size |
| 23 | PRJNA942288 | SRP426297 | Bad files |
| 24 | PRJNA716307 | SRP311668 | Analyzed |
| 25 | PRJNA963864 | SRP435229 | Analyzed |
| 26 | PRJNA954393 | SRP432040 | Small data size |
| 27 | PRJNA954394 | SRP432025 | Small data size |
| 28 | PRJNA954387 | SRP432023 | Small data size |
| 29 | PRJNA1084816 | SRP493680 | Analyzed |
| 30 | PRJNA943485 | SRP426819 | scRNA-seq |
| 31 | PRJNA1077310 | SRP490054 | Analyzed |
| 32 | PRJNA944104 | SRP426965 | Small data size |
| 33 | PRJNA1026649 | SRP465515 | scRNA-seq |
| 34 | PRJNA1030039 | SRP467417 | Small data size |
| 35 | PRJNA1055881 | SRP479631 | Analyzed |
| 36 | PRJNA1069047 | SRP485934 | Analyzed |
| 37 | PRJNA1029528 | SRP467025 | Analyzed |
| 38 | PRJNA1021496 | SRP463376 | Analyzed |
| 39 | PRJNA1048460 | SRP475792 | Small data size |
| 40 | PRJNA898342 | SRP406323 | Analyzed |
| 41 | PRJNA1041715 | SRP472503 | Only three samples |
| 42 | PRJNA1008683 | SRP456765 | Analyzed |
| 43 | PRJNA770265 | SRP340820 | ATAC-seq+RNA-Seq |
| 44 | PRJNA1032114 | SRP468431 | Small data size |
| 45 | PRJNA1023286 | SRP464214 | Analyzed |
| 46 | PRJNA1023284 | SRP464207 | Analyzed |
| 47 | PRJNA1023283 | SRP464206 | ATAC-seq |

| | | | |
|---|---|---|---|
| 48 | PRJNA938432 | SRP424435 | Analyzed |
| 49 | PRJNA945061 | SRP427519 | Small data size |
| 50 | PRJNA945057 | SRP427910 | Hi-C |
| 51 | PRJNA945055 | SRP427525 | Small data size |
| 52 | PRJNA888986 | SRP401802 | Analyzed |
| 53 | PRJNA995518 | SRP449844 | Analyzed |
| 54 | PRJNA934309 | SRP422257 | Small data size |
| 55 | PRJNA993693 | SRP448630 | Analyzed |
| 56 | PRJNA907148 | SRP410755 | scRNA-seq |
| 57 | PRJNA907147 | SRP410752 | Small data size |
| 58 | PRJNA884479 | SRP399763 | snRNA-seq |
| 59 | PRJNA1005927 | SRP455344 | snRNA-seq |
| 60 | PRJNA976199 | SRP439503 | Small data size |
| 61 | PRJNA901181 | SRP407665 | Analyzed |
| 62 | PRJNA994932 | SRP449642 | nascent RNA-Seq |
| 63 | PRJNA849203 | SRP380203 | Small data size |
| 64 | PRJNA894586 | SRP404717 | RNA-seq+ATAC-seq |
| 65 | PRJNA872400 | SRP393496 | Analyzed |
| 66 | PRJNA903187 | SRP408641 | Analyzed |
| 67 | PRJNA966986 | SRP435792 | Analyzed |
| 68 | PRJNA949157 | SRP429490 | snRNA-seq |
| 69 | PRJNA926937 | SRP418840 | Small data size |
| 70 | PRJNA903139 | SRP408616 | snRNA-seq |
| 71 | PRJNA950953 | SRP430326 | Small data size |
| 72 | PRJNA954305 | SRP431956 | Analyzed |
| 73 | PRJNA961771 | SRP434433 | Analyzed |
| 74 | PRJNA918693 | SRP416052 | Analyzed |
| 75 | PRJNA876264 | SRP395415 | scRNA-seq+ATAC-seq |
| 76 | PRJNA913945 | SRP413931 | Analyzed |
| 77 | PRJNA879408 | SRP396719 | Analyzed |
| 78 | PRJNA882769 | SRP398407 | Analyzed |
| 79 | PRJNA930761 | SRP420701 | Analyzed |
| 80 | PRJNA903160 | SRP408610 | Analyzed |
| 81 | PRJNA926996 | SRP418896 | Only three samples |
| 82 | PRJNA899001 | SRP406609 | Analyzed |
| 83 | PRJNA817159 | SRP364658 | scRNA-seq |
| 84 | PRJNA785586 | SRP348932 | snRNA-seq |
| 85 | PRJNA851378 | SRP382880 | Analyzed |
| 86 | PRJNA850399 | SRP382287 | scRNA-seq |
| 87 | PRJNA746567 | SRP328331 | Analyzed |
| 88 | PRJNA892043 | SRP403451 | Analyzed |
| 89 | PRJNA890288 | SRP405319 | Analyzed |
| 90 | PRJNA874744 | SRP394762 | Analyzed |
| 91 | PRJNA880333 | SRP396982 | Analyzed |

| | | | |
|---|---|---|---|
| 92 | PRJNA767567 | SRP339495 | scRNA-seq |
| 93 | PRJNA807643 | SRP360156 | Analyzed |
| 94 | PRJNA807644 | SRP360159 | Analyzed |
| 95 | PRJNA700664 | SRP305366 | Analyzed |
| 96 | PRJNA758929 | SRP334731 | Analyzed |
| 97 | PRJNA830771 | SRP371712 | Analyzed |
| 98 | PRJNA782006 | SRP346962 | scRNA-seq |
| 99 | PRJNA851593 | SRP383012 | Small data size |
| 100 | PRJNA691543 | SRP301422 | Small data size |
| 101 | PRJNA691544 | SRP301423 | Analyzed |
| 102 | PRJNA847625 | SRP379420 | Small data size |
| 103 | PRJNA839695 | SRP376103 | Small data size |
| 104 | PRJNA839696 | SRP376094 | Small data size |
| 105 | PRJNA795162 | SRP353806 | Small data size |
| 106 | PRJNA729044 | SRP319378 | Small data size |
| 107 | PRJNA827611 | SRP370701 | Analyzed |
| 108 | PRJNA821860 | SRP366930 | Analyzed |
| 109 | PRJNA813409 | SRP362827 | snRNA-seq |
| 110 | PRJNA813410 | SRP362822 | Analyzed |
| 111 | PRJNA811136 | SRP361821 | Small data size |
| 112 | PRJNA705144 | SRP308358 | Small data size |
| 113 | PRJNA763179 | SRP337022 | Small data size |
| 114 | PRJNA753885 | SRP332144 | Small data size |
| 115 | PRJNA691343 | SRP301318 | Analyzed |
| 116 | PRJNA690788 | SRP300905 | Analyzed |
| 117 | PRJNA673420 | SRP290168 | scRNA-seq |
| 118 | PRJNA771360 | SRP341403 | Analyzed |
| 119 | PRJNA757728 | SRP334090 | snRNA-seq |
| 120 | PRJNA687763 | SRP299450 | Small data size |
| 121 | PRJNA707410 | SRP309692 | Small data size |
| 122 | PRJNA707411 | SRP309689 | Analyzed |
| 123 | PRJNA707412 | SRP309690 | Small data size |
| 124 | PRJNA715728 | SRP311342 | Small data size |
| 125 | PRJNA752877 | SRP331528 | Analyzed |
| 126 | PRJNA753833 | SRP332095 | Analyzed |
| 127 | PRJNA742071 | SRP325901 | Small data size |
| 128 | PRJNA728744 | SRP319261 | Small data size |
| 129 | PRJNA728741 | SRP319262 | Analyzed |
| 130 | PRJNA729416 | SRP319427 | Analyzed |
| 131 | PRJNA739973 | SRP325058 | Analyzed |
| 132 | PRJNA746403 | SRP328337 | Small data size |
| 133 | PRJNA667096 | SRP286213 | Analyzed |
| 134 | PRJNA723599 | SRP315768 | scRNA-seq |
| 135 | PRJNA723598 | SRP315767 | scRNA-seq |

| | | | |
|---|---|---|---|
| 136 | PRJNA716677 | SRP311860 | Analyzed |
| 137 | PRJNA706199 | SRP309056 | Small data size |
| 138 | PRJNA644445 | SRP270410 | scRNA-seq |
| 139 | PRJNA694049 | SRP302849 | Small data size |
| 140 | PRJNA664122 | SRP282921 | Small data size |
| 141 | PRJNA691970 | SRP301650 | Small data size |
| 142 | PRJNA483460 | SRP155744 | Small data size |
| 143 | PRJNA685410 | SRP298006 | Analyzed |
| 144 | PRJNA657012 | SRP277374 | scRNA-seq |
| 145 | PRJNA602538 | SRP243780 | Small data size |
| 146 | PRJNA627727 | SRP258168 | Analyzed |
| 147 | PRJNA667280 | SRP286323 | Analyzed |
| 148 | PRJNA615366 | SRP254109 | scRNA-seq |
| 149 | PRJNA608711 | SRP250704 | Analyzed |
| 150 | PRJNA598680 | SRP239316 | scRNA-seq |
| 151 | PRJNA598660 | SRP239306 | scRNA-seq |
| 152 | PRJNA631902 | SRP261247 | scRNA-seq |
| 153 | PRJNA602299 | SRP243446 | snRNA-seq |
| 154 | PRJNA474958 | SRP149946 | Small data size |
| 155 | PRJNA518104 | SRP183070 | Small data size |
| 156 | PRJNA588788 | SRP229494 | Analyzed |
| 157 | PRJNA553523 | SRP213880 | Small data size |
| 158 | PRJNA540830 | SRP194567 | scRNA-seq |
| 159 | PRJNA511441 | SRP174208 | Small data size |
| 160 | PRJNA556201 | SRP216247 | Small data size |
| 161 | PRJNA556200 | SRP216250 | Small data size |
| 162 | PRJNA525951 | SRP187821 | Small data size |
| 163 | PRJNA525702 | SRP187600 | Small data size |
| 164 | PRJNA533562 | SRP192961 | Small data size |
| 165 | PRJNA449748 | SRP139606 | ATAC-seq+ChIP-Seq+ChIP-Seq |
| 166 | PRJNA360850 | SRP096370 | Small data size |
| 167 | PRJNA472064 | SRP148460 | Small data size |
| 168 | PRJNA510760 | SRP173901 | Analyzed |
| 169 | PRJNA489079 | SRP159463 | Analyzed |
| 170 | PRJNA401661 | SRP131333 | Small data size |
| 171 | PRJNA486277 | SRP158112 | Small data size |
| 172 | PRJNA419639 | SRP125551 | Analyzed |
| 173 | PRJNA412500 | SRP119064 | Small data size |
| 174 | PRJNA413371 | SRP119455 | Small data size |
| 175 | PRJNA401847 | SRP124513 | scRNA-seq |
| 176 | PRJNA362540 | SRP097094 | Analyzed |
| 177 | PRJNA344779 | SRP090566 | Small data size |
| 178 | PRJNA294612 | SRP063269 | Small data size |
| 179 | PRJNA387078 | SRP107340 | ChIP-Seq |

| | | | |
|---|---|---|---|
| 180 | PRJNA387079 | SRP107339 | scRNA-seq |
| 181 | PRJNA356884 | SRP094853 | Small data size |
| 182 | PRJNA310593 | SRP069184 | Small data size |
| 183 | PRJNA329416 | SRP078692 | Small data size |
| 184 | PRJNA269580 | SRP050896 | sRNA-Seq |
| 185 | PRJNA235922 | SRP035523 | Analyzed |
| 186 | PRJNA226225 | SRP032499 | Small data size |
| 187 | PRJNA185990 | SRP017849 | Small data size |

The datasets (highlighted in yellow color) were analyzed in the present study. The raw data for these datasets are available in the NCBI SRA database and can be retrieved using the respective project accession numbers (Accs) and raw data Accs.

**Table S2. PacBio cDNA data**

| | Cell ID | Tissue | Size | Total | APP | APP/total |
|---|---|---|---|---|---|---|
| 1 | m140119_095807_sherri_c100625400310000001823112208061413 | Brain | 1-2kb | 42192 | 16 | 0.04% |
| 2 | m140220_010916_42141_c100623532550000001823111708061436 | Brain | 1-2kb | 51426 | 47 | 0.09% |
| 3 | m140220_042756_42141_c100623532550000001823111708061437 | Brain | 1-2kb | 48309 | 40 | 0.08% |
| 4 | m140220_074709_42141_c100633500150000001823121009121490 | Brain | 1-2kb | 25333 | 21 | 0.08% |
| 5 | m140225_212801_42141_c100631202390000001823105309121430 | Brain | 1-2kb | 39678 | 28 | 0.07% |
| 6 | m140226_004703_42141_c100631202390000001823105309121431 | Brain | 1-2kb | 80161 | 64 | 0.08% |
| 7 | m140226_040616_42141_c100631202390000001823105309121432 | Brain | 1-2kb | 31473 | 26 | 0.08% |
| 8 | m140119_132554_sherri_c100625400310000001823112208061414 | Brain | 2-3kb | 21118 | 11 | 0.05% |
| 9 | m140119_165149_sherri_c100623990070000001823119508061480 | Brain | 2-3kb | 30111 | 33 | 0.11% |
| 10 | m140121_032639_42142_c100622940150000001823111308061430 | Brain | 2-3kb | 37763 | 37 | 0.10% |
| 11 | m140121_064858_42142_c100622940150000001823111308061431 | Brain | 2-3kb | 35214 | 26 | 0.07% |
| 12 | m140220_110624_42141_c100633500150000001823121009121491 | Brain | 2-3kb | 20092 | 54 | 0.27% |
| 13 | m140220_142535_42141_c100633500150000001823121009121492 | Brain | 2-3kb | 26382 | 43 | 0.16% |
| 14 | m140220_174448_42141_c100633500150000001823121009121493 | Brain | 2-3kb | 27360 | 58 | 0.21% |
| 15 | m140226_072529_42141_c100631202390000001823105309121433 | Brain | 2-3kb | 12172 | 30 | 0.25% |
| 16 | m140226_104442_42141_c100631202390000001823105309121435 | Brain | 2-3kb | 12946 | 18 | 0.14% |
| 17 | m140226_140355_42141_c100631202390000001823105309121436 | Brain | 2-3kb | 12794 | 21 | 0.16% |
| 18 | m140119_202143_sherri_c100623990070000001823119508061481 | Brain | 3-6kb | 18780 | 2 | 0.01% |
| 19 | m140119_234911_sherri_c100623990070000001823119508061482 | Brain | 3-6kb | 14545 | 1 | 0.01% |
| 20 | m140121_101415_42142_c100622940150000001823111308061432 | Brain | 3-6kb | 15145 | 2 | 0.01% |
| 21 | m140121_133642_42142_c100622940150000001823111308061433 | Brain | 3-6kb | 12862 | 1 | 0.01% |
| 22 | m140213_024332_42142_c100632392550000001823124209121480 | Brain | 3-6kb | 11289 | 4 | 0.04% |
| 23 | m140213_061904_42142_c100632392550000001823124209121481 | Brain | 3-6kb | 11942 | 2 | 0.02% |
| 24 | m140213_095745_42142_c100632392550000001823124209121482 | Brain | 3-6kb | 16275 | 3 | 0.02% |
| 25 | m140213_133714_42142_c100632392550000001823124209121483 | Brain | 3-6kb | 14865 | 6 | 0.04% |
| 26 | m140206_031640_42141_c110038252550000001823107706241420 | Brain | 5-10kb | 410 | 0 | 0.00% |
| 27 | m140206_063529_42141_c110038252550000001823107706241421 | Brain | 5-10kb | 324 | 0 | 0.00% |
| 28 | m140206_095437_42141_c110038252550000001823107706241422 | Brain | 5-10kb | 449 | 0 | 0.00% |

| | | | | | | |
|---|---|---|---|---|---|---|
| 29 | m140206_131350_42141_c110038252550000001823107706241423 | Brain | 5-10kb | 311 | 0 | 0.00% |
| 30 | m140206_163303_42141_c110038252550000001823107706241424 | Brain | 5-10kb | 256 | 0 | 0.00% |
| 31 | m140206_195216_42141_c110038252550000001823107706241425 | Brain | 5-10kb | 214 | 0 | 0.00% |
| 32 | m140213_023318_sherri_c110038432550000001823107706241480 | Brain | 5-10kb | 115 | 0 | 0.00% |
| 33 | m140213_055450_sherri_c110038432550000001823107706241481 | Brain | 5-10kb | 235 | 0 | 0.00% |
| 34 | m140213_091856_sherri_c110038432550000001823107706241482 | Brain | 5-10kb | 307 | 0 | 0.00% |
| 35 | m140213_124252_sherri_c110038432550000001823107706241483 | Brain | 5-10kb | 101 | 0 | 0.00% |
| 36 | m140213_160622_sherri_c110038432550000001823107706241484 | Brain | 5-10kb | 237 | 0 | 0.00% |
| 37 | m140213_192933_sherri_c110038432550000001823107706241485 | Brain | 5-10kb | 43 | 0 | 0.00% |
| 38 | m140308_033548_sherri_c100641472550000001823126010151460 | Brain | 5-10kb | 173 | 0 | 0.00% |
| 39 | m140308_070501_sherri_c100641472550000001823126010151461 | Brain | 5-10kb | 6175 | 0 | 0.00% |
| 40 | m140308_104027_sherri_c100641472550000001823126010151462 | Brain | 5-10kb | 7909 | 0 | 0.00% |
| 41 | m140308_141259_sherri_c100641472550000001823126010151463 | Brain | 5-10kb | 8722 | 0 | 0.00% |
| 42 | m140308_174717_sherri_c100641472550000001823126010151464 | Brain | 5-10kb | 7884 | 1 | 0.01% |
| 43 | m140308_212337_sherri_c100641472550000001823126010151465 | Brain | 5-10kb | 10947 | 0 | 0.00% |
| 44 | m140309_005951_sherri_c100641472550000001823126010151466 | Brain | 5-10kb | 10435 | 1 | 0.01% |
| 45 | m140309_043201_sherri_c100641472550000001823126010151467 | Brain | 5-10kb | 6977 | 0 | 0.00% |
| 46 | m140517_022630_42141_c110042412550000001823111106241444 | Brain | 5-10kb | 13264 | 0 | 0.00% |
| 47 | m140517_064855_42141_c110042412550000001823111106241445 | Brain | 5-10kb | 10795 | 0 | 0.00% |
| 48 | m140518_045655_42141_c110034182550000001823102605301486 | Heart | 5-10kb | 4178 | 0 | 0.00% |
| 49 | m140518_092242_42141_c110034182550000001823102605301487 | Heart | 5-10kb | 5969 | 0 | 0.00% |
| 50 | m140121_032645_42141_c100626750070000001823119808061460 | Heart | 1-2kb | 33392 | 1 | 0.00% |
| 51 | m140121_064408_42141_c100626750070000001823119808061461 | Heart | 1-2kb | 28608 | 1 | 0.00% |
| 52 | m140131_031135_42141_c110038482550000001823107706241430 | Heart | 1-2kb | 6902 | 0 | 0.00% |
| 53 | m140131_063020_42141_c110038482550000001823107706241431 | Heart | 1-2kb | 4272 | 0 | 0.00% |
| 54 | m140131_095235_42141_c110038482550000001823107706241432 | Heart | 1-2kb | 5090 | 0 | 0.00% |
| 55 | m140131_131341_42141_c110038482550000001823107706241433 | Heart | 1-2kb | 4150 | 0 | 0.00% |
| 56 | m140221_003203_42141_c100634362510000001823117809121430 | Heart | 1-2kb | 32514 | 1 | 0.00% |
| 57 | m140221_035026_42141_c100634362510000001823117809121431 | Heart | 1-2kb | 36488 | 4 | 0.01% |
| 58 | m140221_070939_42141_c100634362510000001823117809121433 | Heart | 1-2kb | 31503 | 3 | 0.01% |

| | | | | | | |
|---|---|---|---|---|---|---|
| 59 | m140121_100730_42141_c100626750070000001823119808061462 | Heart | 2-3kb | 24404 | 1 | 0.00% |
| 60 | m140121_132657_42141_c100626060070000001823118408061490 | Heart | 2-3kb | 19963 | 2 | 0.01% |
| 61 | m140221_102852_42141_c100634362510000001823117809121434 | Heart | 2-3kb | 17033 | 20 | 0.12% |
| 62 | m140221_134805_42141_c100634362510000001823117809121435 | Heart | 2-3kb | 17176 | 15 | 0.09% |
| 63 | m140221_170718_42141_c100634362510000001823117809121436 | Heart | 2-3kb | 18020 | 16 | 0.09% |
| 64 | m140121_164721_42141_c100626060070000001823118408061491 | Heart | 3-6kb | 7323 | 1 | 0.01% |
| 65 | m140121_200633_42141_c100626060070000001823118408061492 | Heart | 3-6kb | 12368 | 3 | 0.02% |
| 66 | m140220_222848_42142_c100634462490000001823117809121470 | Heart | 3-6kb | 7682 | 1 | 0.01% |
| 67 | m140221_015407_42142_c100634462490000001823117809121473 | Heart | 3-6kb | 9217 | 0 | 0.00% |
| 68 | m140221_052041_42142_c100634462490000001823117809121474 | Heart | 3-6kb | 11016 | 0 | 0.00% |
| 69 | m140309_102439_sherri_c100641502550000001823126010151400 | Heart | 3-6kb | 26777 | 4 | 0.01% |
| 70 | m140309_135823_sherri_c100641502550000001823126010151401 | Heart | 3-6kb | 27509 | 6 | 0.02% |
| 71 | m140309_173351_sherri_c100641502550000001823126010151402 | Heart | 3-6kb | 21773 | 3 | 0.01% |
| 72 | m140309_211055_sherri_c100641502550000001823126010151403 | Heart | 3-6kb | 22165 | 3 | 0.01% |
| 73 | m140221_084430_42142_c100634462490000001823117809121475 | Heart | 5-10kb | 809 | 0 | 0.00% |
| 74 | m140221_120945_42142_c100634462490000001823117809121476 | Heart | 5-10kb | 745 | 0 | 0.00% |
| 75 | m140221_153518_42142_c100634462490000001823117809121477 | Heart | 5-10kb | 815 | 0 | 0.00% |
| 76 | m140310_004313_sherri_c100641502550000001823126010151404 | Heart | 5-10kb | 8521 | 0 | 0.00% |
| 77 | m140310_041954_sherri_c100641502550000001823126010151405 | Heart | 5-10kb | 8074 | 0 | 0.00% |
| 78 | m140310_080044_sherri_c100641502550000001823126010151406 | Heart | 5-10kb | 7537 | 0 | 0.00% |
| 79 | m140310_113630_sherri_c100641502550000001823126010151407 | Heart | 5-10kb | 5729 | 0 | 0.00% |
| 80 | m140220_223157_sherri_c100633820630000001823124709121450 | Liver | 1-2kb | 38505 | 4 | 0.01% |
| 81 | m140221_015622_sherri_c100633820630000001823124709121451 | Liver | 1-2kb | 36045 | 3 | 0.01% |
| 82 | m140221_052005_sherri_c100633820630000001823124709121452 | Liver | 1-2kb | 30866 | 1 | 0.00% |
| 83 | m140320_000816_sherri_c110027181910000001823097005201470 | Liver | 1-2kb | 31752 | 4 | 0.01% |
| 84 | m140320_033434_sherri_c110027181910000001823097005201471 | Liver | 1-2kb | 34985 | 5 | 0.01% |
| 85 | m140326_032433_42141_c110038412550000001823107706241403 | Liver | 1-2kb | 4088 | 0 | 0.00% |
| 86 | m140326_064556_42141_c110038412550000001823107706241404 | Liver | 1-2kb | 4149 | 0 | 0.00% |
| 87 | m140326_100642_42141_c110038412550000001823107706241405 | Liver | 1-2kb | 4290 | 0 | 0.00% |
| 88 | m140408_022527_sherri_c110020411270000001823099305201403 | Liver | 1-2kb | 33642 | 5 | 0.01% |

| | | | | | | |
|---|---|---|---|---|---|---|
| 89 | m140408_054526_sherri_c110020411270000001823099305201404 | Liver | 1-2kb | 33273 | 5 | 0.02% |
| 90 | m140408_090610_sherri_c110020411270000001823099305201405 | Liver | 1-2kb | 43859 | 3 | 0.01% |
| 91 | m140221_084347_sherri_c100633820630000001823124709121453 | Liver | 2-3kb | 17053 | 4 | 0.02% |
| 92 | m140221_120940_sherri_c100633820630000001823124709121454 | Liver | 2-3kb | 28372 | 5 | 0.02% |
| 93 | m140221_153431_sherri_c100633820630000001823124709121455 | Liver | 2-3kb | 16529 | 4 | 0.02% |
| 94 | m140320_070202_sherri_c110027181910000001823097005201472 | Liver | 2-3kb | 30944 | 7 | 0.02% |
| 95 | m140320_103039_sherri_c110027181910000001823097005201473 | Liver | 2-3kb | 31499 | 7 | 0.02% |
| 96 | m140326_132650_42141_c110038412550000001823107706241406 | Liver | 2-3kb | 1938 | 0 | 0.00% |
| 97 | m140326_164643_42141_c110038412550000001823107706241407 | Liver | 2-3kb | 2192 | 0 | 0.00% |
| 98 | m140327_214046_sherri_c110045012490000001823114106241490 | Liver | 2-3kb | 6291 | 1 | 0.02% |
| 99 | m140328_011325_sherri_c110045012490000001823114106241493 | Liver | 2-3kb | 4380 | 0 | 0.00% |
| 100 | m140408_022216_42141_c110031780070000001823100905301410 | Liver | 2-3kb | 33695 | 3 | 0.01% |
| 101 | m140408_054129_42141_c110031780070000001823100905301411 | Liver | 2-3kb | 34401 | 7 | 0.02% |
| 102 | m140408_090042_42141_c110031780070000001823100905301412 | Liver | 2-3kb | 27589 | 5 | 0.02% |
| 103 | m140225_213446_42142_c100631882230000001823120209121480 | Liver | 3-6kb | 18557 | 8 | 0.04% |
| 104 | m140226_005728_42142_c100631882230000001823120209121481 | Liver | 3-6kb | 16612 | 10 | 0.06% |
| 105 | m140226_042308_42142_c100631882230000001823120209121482 | Liver | 3-6kb | 9780 | 4 | 0.04% |
| 106 | m140226_074514_42142_c100631882230000001823120209121483 | Liver | 3-6kb | 13016 | 3 | 0.02% |
| 107 | m140226_110708_42142_c100631882230000001823120209121484 | Liver | 3-6kb | 10722 | 6 | 0.06% |
| 108 | m140320_135907_sherri_c110027181910000001823097005201474 | Liver | 3-6kb | 6853 | 2 | 0.03% |
| 109 | m140320_172449_sherri_c110027181910000001823097005201475 | Liver | 3-6kb | 12732 | 3 | 0.02% |
| 110 | m140328_044318_sherri_c110045012490000001823114106241494 | Liver | 3-6kb | 6764 | 1 | 0.01% |
| 111 | m140328_081253_sherri_c110045012490000001823114106241495 | Liver | 3-6kb | 8544 | 2 | 0.02% |
| 112 | m140328_114130_sherri_c110045012490000001823114106241496 | Liver | 3-6kb | 6685 | 3 | 0.04% |
| 113 | m140328_151420_sherri_c110045012490000001823114106241497 | Liver | 3-6kb | 6870 | 1 | 0.01% |
| 114 | m140408_122301_42141_c110030790010000001823106505301450 | Liver | 3-6kb | 17602 | 4 | 0.02% |
| 115 | m140408_122608_sherri_c110020411270000001823099305201406 | Liver | 3-6kb | 18906 | 3 | 0.02% |
| 1 | m140731_222056_42161_c100698070630000001823143403261500 | MCF-7 | 1-2kb | 14412 | 0 | 0.00% |
| 2 | m140801_014337_42161_c100698070630000001823143403261501 | MCF-7 | 1-2kb | 15693 | 0 | 0.00% |
| 3 | m140801_050734_42161_c100698070630000001823143403261502 | MCF-7 | 1-2kb | 15936 | 0 | 0.00% |

| | | | | | | |
|---|---|---|---|---|---|---|
| 4 | m140801_082952_42161_c100698070630000001823143403261503 | MCF-7 | 3-4kb | 17504 | 5 | 0.03% |
| 5 | m140801_115238_42161_c100698070630000001823143403261504 | MCF-7 | 3-4kb | 35587 | 27 | 0.08% |
| 6 | m140801_151509_42161_c100698070630000001823143403261505 | MCF-7 | 3-4kb | 33837 | 32 | 0.09% |
| 7 | m140804_215651_42141_c100700040630000001823139203261500 | MCF-7 | 5-6kb | 16611 | 1 | 0.01% |
| 8 | m140805_011552_42141_c100700040630000001823139203261501 | MCF-7 | 5-6kb | 17584 | 4 | 0.02% |
| 9 | m140805_044156_42141_c100700040630000001823139203261502 | MCF-7 | 7-8kb | 25329 | 0 | 0.00% |
| 10 | m140805_080756_42141_c100700040630000001823139203261503 | MCF-7 | 7-8kb | 27540 | 3 | 0.01% |
| 11 | m140805_113513_42141_c100700040630000001823139203261504 | MCF-7 | 9-10kb | 36711 | 2 | 0.01% |
| 12 | m140805_150259_42141_c100700040630000001823139203261505 | MCF-7 | 9-10kb | 35107 | 2 | 0.01% |
| 13 | m140808_221025_42161_c100696951270000001823138003261560 | MCF-7 | 1-2kb | 11898 | 0 | 0.00% |
| 14 | m140809_012938_42161_c100696951270000001823138003261561 | MCF-7 | 1-2kb | 6540 | 1 | 0.02% |
| 15 | m140809_044851_42161_c100696951270000001823138003261562 | MCF-7 | 1-2kb | 12614 | 0 | 0.00% |
| 16 | m140809_080804_42161_c100696951270000001823138003261563 | MCF-7 | 1-2kb | 10106 | 1 | 0.01% |
| 17 | m140809_112717_42161_c100696951270000001823138003261564 | MCF-7 | 3-4kb | 34718 | 22 | 0.06% |
| 18 | m140809_144702_42161_c100696951270000001823138003261565 | MCF-7 | 3-4kb | 33866 | 20 | 0.06% |
| 19 | m140809_181052_42161_c100696951270000001823138003261566 | MCF-7 | 3-4kb | 33146 | 25 | 0.08% |
| 20 | m140809_213120_42161_c100696870310000001823138003261570 | MCF-7 | 3-4kb | 26520 | 14 | 0.05% |
| 21 | m140810_025821_42161_c100696870310000001823138003261571 | MCF-7 | 5-6kb | 39779 | 12 | 0.03% |
| 22 | m140810_061610_42161_c100696870310000001823138003261572 | MCF-7 | 5-6kb | 35920 | 7 | 0.02% |
| 23 | m140810_093523_42161_c100696870310000001823138003261573 | MCF-7 | 5-6kb | 39665 | 7 | 0.02% |
| 24 | m140810_125744_42161_c100696870310000001823138003261574 | MCF-7 | 7-8kb | 46524 | 1 | 0.00% |
| 25 | m140810_161642_42161_c100693060150000001823146703241590 | MCF-7 | 7-8kb | 40087 | 4 | 0.01% |
| 26 | m140810_193644_42161_c100693060150000001823146703241591 | MCF-7 | 7-8kb | 45641 | 5 | 0.01% |
| 27 | m140810_225944_42161_c100693060150000001823146703241592 | MCF-7 | 9-10kb | 36151 | 0 | 0.00% |
| 28 | m140811_021934_42161_c100693060150000001823146703241593 | MCF-7 | 9-10kb | 37537 | 1 | 0.00% |

Data files from MCF-7 cell samples were downloaded from http://datasets.pacb.com.s3.amazonaws.com/2013/IsoSeqHumanMCF7Transcriptome/list.html and and those from 115 human brain, heart, and liver tissues were downloaded from http://datasets.pacb.com.s3.amazonaws.com/2014/Iso-seq_Human_Tissues/list.html. Total represents the total number of CCSs (Circular Consensus Sequences); Size represents the library size for PacBio full-length transcriptom sequencing; APP represents the number of CCSs aligned to APP transcripts.

**Table S3. PCR primers to confirm the decrease in the usage of APP/58417N**

| Target | Forward primer | Reverse primer | AT |
|---|---|---|---|
| APP/58417N | ATCCCACTCGCACAGCAGC | CATGTTCAGTCTGCCACAGAACATGG | 58 |
| APP/36594N | GAACTGCAGATCACCAATGTGG | AGGGCATCACTTACAAACTCACC | 58 |

The primer for reverse transcription (RT) has a concentration of 1 μM, and the PCR primer concentration is 1 μM. AT: annealing temperature. For each target (APP/58417N or APP/36594N), the qPCR amplification was repeated three times to produce an average cycle-threshold (Ct) value for the U1 snRNA over-expressing group and the control group using hESC-derived neurons, respectively. Relative abundance was calculated with the ΔCt method (APP-58417N vs APP-36594N), and log2 fold-changes of the mean Ct values were compared between groups by unpaired two-tailed t-tests; $p < 0.05$ was considered significant.

**Table S4. The DGEA result of GSE124951**

| **Gene or LncRNA** | **logFC** | **logCPM** | **PValue** | **FDR** |
|---|---|---|---|---|
| IQCJ-SCHIP1 | 9.97 | 1.88 | 1.60E-16 | 4.06E-12 |
| SCUBE3 | 2.78 | 6.28 | 2.76E-15 | 3.49E-11 |
| H4C11 | 9.68 | 1.59 | 1.15E-13 | 9.67E-10 |
| ID3 | 2.75 | 6.65 | 1.67E-13 | 1.06E-09 |
| ADA | -1.87 | 6.42 | 5.39E-13 | 2.73E-09 |
| H4C15 | 10.88 | 2.76 | 5.54E-12 | 2.33E-08 |
| ID1 | 2.34 | 7.19 | 1.14E-11 | 4.13E-08 |
| ENSG00000259002 | 5.71 | 1.79 | 1.89E-10 | 5.89E-07 |
| FTH1 | -1.62 | 12.58 | 2.28E-10 | 5.89E-07 |
| ENSG00000264545 | -9.20 | 1.14 | 2.33E-10 | 5.89E-07 |
| DDIT4 | -1.56 | 6.22 | 2.80E-10 | 6.44E-07 |
| L1CAM | -1.68 | 5.25 | 3.23E-10 | 6.54E-07 |
| MMP11 | -1.86 | 6.34 | 3.40E-10 | 6.54E-07 |
| MEDAG | 1.94 | 5.82 | 3.62E-10 | 6.54E-07 |
| ENSG00000269972 | -9.11 | 1.05 | 1.24E-09 | 2.08E-06 |
| LTBP1 | 1.75 | 6.44 | 1.82E-09 | 2.88E-06 |
| MAN1C1 | -1.57 | 5.70 | 2.30E-09 | 3.41E-06 |
| TP53I11 | -1.72 | 7.24 | 4.01E-09 | 5.63E-06 |
| KLRK1 | -2.62 | 3.21 | 5.68E-09 | 7.56E-06 |
| GAP43 | 3.63 | 3.96 | 6.04E-09 | 7.64E-06 |
| ENSG00000306517 | -2.02 | 5.19 | 9.58E-09 | 1.15E-05 |
| FLT1 | 2.30 | 3.31 | 1.38E-08 | 1.59E-05 |
| ENSG00000267143 | 8.81 | 0.77 | 1.59E-08 | 1.70E-05 |
| TM7SF2 | -1.66 | 4.64 | 1.62E-08 | 1.70E-05 |
| IFI30 | -1.57 | 5.46 | 2.06E-08 | 2.07E-05 |
| ENSG00000267228 | 8.82 | 0.78 | 2.14E-08 | 2.07E-05 |
| ENSG00000305069 | -8.99 | 0.94 | 2.21E-08 | 2.07E-05 |
| TAGLN2 | 1.55 | 7.30 | 2.64E-08 | 2.39E-05 |

| | | | | |
|---|---|---|---|---|
| ENSG00000295507 | -8.97 | 0.92 | 4.78E-08 | 4.16E-05 |
| ADGRG1 | 2.73 | 2.94 | 4.98E-08 | 4.16E-05 |
| ENSG00000296951 | 8.74 | 0.70 | 5.10E-08 | 4.16E-05 |
| NEAT1 | -1.28 | 8.07 | 7.84E-08 | 6.19E-05 |
| ENSG00000263567 | 8.74 | 0.70 | 1.15E-07 | 8.84E-05 |
| ENSG00000270321 | -2.84 | 2.48 | 1.23E-07 | 9.14E-05 |
| ANPEP | 1.93 | 7.15 | 1.30E-07 | 9.40E-05 |
| PROCR | 1.56 | 5.32 | 1.57E-07 | 1.10E-04 |
| CD44 | 1.41 | 7.30 | 1.63E-07 | 1.10E-04 |
| NOTCH3 | 1.55 | 5.90 | 1.66E-07 | 1.10E-04 |
| ENSG00000298505 | 8.51 | 0.49 | 3.45E-07 | 2.23E-04 |
| ADAM19 | 1.30 | 5.34 | 3.52E-07 | 2.23E-04 |
| ENSG00000307279 | 9.01 | 0.96 | 3.90E-07 | 2.41E-04 |
| SLC12A9-AS1 | -8.48 | 0.46 | 4.17E-07 | 2.51E-04 |
| ENSG00000265393 | 8.47 | 0.46 | 4.83E-07 | 2.84E-04 |
| MFAP2 | 1.30 | 7.83 | 5.13E-07 | 2.95E-04 |
| FTL | -1.19 | 14.00 | 6.01E-07 | 3.38E-04 |
| NTSR1 | 4.51 | 1.56 | 9.04E-07 | 4.97E-04 |
| CARD16 | -1.52 | 4.47 | 9.26E-07 | 4.98E-04 |
| GLIPR1 | 1.48 | 6.34 | 1.03E-06 | 5.43E-04 |
| ENSG00000280800 | -1.39 | 11.55 | 1.06E-06 | 5.45E-04 |
| ACKR3 | -1.32 | 5.11 | 1.10E-06 | 5.58E-04 |
| STX1A | 1.38 | 5.49 | 1.18E-06 | 5.87E-04 |
| CTSH | -1.57 | 4.11 | 1.28E-06 | 6.23E-04 |
| LINC03008 | -8.58 | 0.55 | 1.31E-06 | 6.25E-04 |
| ENSG00000280614 | -1.24 | 11.43 | 1.37E-06 | 6.44E-04 |
| SERPINA1 | -5.00 | 0.90 | 1.65E-06 | 7.45E-04 |
| ENSG00000305635 | -1.25 | 5.30 | 1.65E-06 | 7.45E-04 |
| SAA1 | -9.09 | 1.02 | 2.05E-06 | 9.10E-04 |
| ECSCR | 3.16 | 1.78 | 2.46E-06 | 1.07E-03 |
| ANGPT1 | -1.39 | 5.34 | 2.78E-06 | 1.19E-03 |
| ITGA5 | 1.18 | 8.05 | 3.04E-06 | 1.28E-03 |
| CTSL | -1.39 | 9.44 | 3.11E-06 | 1.29E-03 |
| GPNMB | -1.30 | 5.84 | 3.46E-06 | 1.41E-03 |
| STC1 | 2.42 | 4.71 | 3.65E-06 | 1.47E-03 |
| IFI35 | -1.33 | 5.65 | 4.41E-06 | 1.73E-03 |
| DBP | -2.86 | 2.16 | 4.46E-06 | 1.73E-03 |
| ENSG00000307045 | -8.22 | 0.21 | 4.55E-06 | 1.74E-03 |
| CARMN | 2.60 | 2.59 | 4.79E-06 | 1.81E-03 |
| ENSG00000250615 | -8.18 | 0.18 | 5.22E-06 | 1.94E-03 |
| ENSG00000285723 | -12.09 | 3.96 | 6.94E-06 | 2.51E-03 |
| LOX | 1.07 | 9.22 | 7.03E-06 | 2.51E-03 |
| TFRC | -1.49 | 4.51 | 7.05E-06 | 2.51E-03 |
| LINC03056 | 8.57 | 0.54 | 7.25E-06 | 2.54E-03 |

| | | | | |
|---|---|---|---|---|
| LCNL1 | -4.05 | 1.24 | 8.82E-06 | 3.05E-03 |
| TAPBPL | -1.33 | 4.21 | 1.17E-05 | 4.00E-03 |
| TAGLN | 1.94 | 7.55 | 1.31E-05 | 4.40E-03 |
| HTR2B | -1.13 | 6.01 | 1.38E-05 | 4.57E-03 |
| SLC25A29 | -1.30 | 4.22 | 1.40E-05 | 4.60E-03 |
| LYPLAL1 | -1.33 | 4.36 | 1.43E-05 | 4.64E-03 |
| SBF2-AS1 | -2.00 | 4.02 | 1.49E-05 | 4.74E-03 |
| LINC01551 | -6.97 | 0.05 | 1.50E-05 | 4.74E-03 |
| ADAMTSL1 | 1.48 | 5.64 | 1.70E-05 | 5.30E-03 |
| ENSG00000261602 | -1.85 | 3.59 | 1.88E-05 | 5.79E-03 |
| NET1 | 1.73 | 3.36 | 1.99E-05 | 6.05E-03 |
| ENSG00000267765 | -8.16 | 0.17 | 2.02E-05 | 6.07E-03 |
| SLC6A6 | 1.39 | 4.39 | 2.06E-05 | 6.13E-03 |
| PRSS35 | 2.08 | 5.07 | 2.25E-05 | 6.63E-03 |
| ENSG00000301732 | 7.96 | 0.00 | 2.38E-05 | 6.91E-03 |
| GNPDA1 | -1.06 | 5.91 | 2.53E-05 | 7.27E-03 |
| GPR68 | 1.35 | 4.07 | 2.62E-05 | 7.45E-03 |
| GPER1 | 1.12 | 5.17 | 2.67E-05 | 7.50E-03 |
| H3C4 | 1.95 | 2.91 | 2.70E-05 | 7.52E-03 |
| TUBB3 | 1.17 | 7.75 | 2.81E-05 | 7.72E-03 |
| ENSG00000289871 | -1.97 | 3.08 | 2.89E-05 | 7.87E-03 |
| SPHK1 | 1.85 | 3.91 | 3.04E-05 | 8.17E-03 |
| KCNK15 | -1.81 | 4.07 | 3.08E-05 | 8.19E-03 |
| ENSG00000283375 | -7.92 | -0.05 | 3.14E-05 | 8.27E-03 |
| SERPINE2 | 1.50 | 8.58 | 3.38E-05 | 8.82E-03 |
| KLF9 | -2.28 | 2.38 | 3.44E-05 | 8.88E-03 |
| CDH13 | 1.24 | 4.65 | 3.51E-05 | 8.95E-03 |
| ENSG00000281181 | -1.01 | 11.49 | 3.85E-05 | 9.73E-03 |

The gene expression matrix of SRP178463, generated with annotations against Ensembl release 114, is available in the NCBI GEO database under the Series accession number GSE124951. The DGEA of GSE124951 identified a set of 48 up-regulated and 52 down-regulated genes or lncRNAs from a total of 25,283 protein-coding genes and lncRNAs. The significantly DE genes were selected according to the criteria |log2foldchange| > 1 and p value<0.01. The expression of *NOTCH1* and *HES1* was also up-regulated by 1.12- and 1.64-fold, with P-values of 0.47 and 0.09, respectively.

**Table S5. Exons and introns of 10 isoforms in Figure 1B**

| S1 | S2 | S3 | S4 | S5 | S6 | S7 | S8 | S9 | S10 |
|---|---|---|---|---|---|---|---|---|---|
| E184 | E184 | E207 | E207 | E207 | E207 | E144 | E180 | | |
| | | | | | | | I41847 | | |
| I80491 | I80491 | I58417 | I58417 | I58417 | I58417 | I58838 | E69 | E308 | |
| | | | | | | | I16501 | I28006 | |
| | | E168a | E168a | E168a | E168a | E168a | E168a | E168a | |

| | | | | | | | | | |
|---|---|---|---|---|---|---|---|---|---|
| | | I21906 | I21906 | I21906 | I21906 | I21906 | I21906 | I21906 | |
| E130 | E130 | E130 | E130 | E130 | E130 | E130 | E130 | E130 | E119 |
| I36594 | I36594 | I36594 | I36594 | I36594 | I36594 | I36594 | I36594 | I36594 | I36594 |
| E113 | E113 | E113 | E113 | E113 | E113 | E113 | E113 | E113 | E113 |
| I2042 | I2042 | I2042 | I2042 | I2042 | I2042 | I2042 | I2042 | I2042 | I2042 |
| E194 | E194 | E194 | E194 | E194 | E194 | E194 | E194 | E194 | E194 |
| I28957 | I28957 | I28957 | I28957 | I28957 | I28957 | I28957 | I28957 | I28957 | I28957 |
| E203 | E203 | E203 | E203 | E203 | E203 | E203 | E203 | E203 | E203 |
| | I21657 | I21657 | | I21657 | I21657 | | | I21657 | I21657 |
| | E168b | E168b | | E168b | E168b | | | E168b | E168b |
| I39362 | I2598 | I2598 | I39362 | | I2598 | I39362 | I39362 | | |
| | E57 | E57 | | I17537 | E57 | | | I17537 | I17537 |
| | I14882 | I14882 | | | I14882 | | | | |
| E134 | E134 | E134 | E134 | E134 | E134 | E134 | E134 | E134 | E134 |
| I6315 | I6315 | I6315 | I6315 | I6315 | I6315 | I6315 | I6315 | I6315 | I6315 |
| E75 | E75 | E75 | E75 | E75 | E75 | E75 | E75 | E75 | E75 |
| I725 | I725 | I725 | I725 | I725 | I725 | I725 | I725 | I725 | I725 |
| E159 | E159 | E159 | E159 | E159 | E159 | E159 | E159 | E159 | E159 |
| I19314 | I19314 | I19314 | I19314 | I19314 | I19314 | I19314 | I19314 | I19314 | I19314 |
| E129 | E129 | E129 | E129 | E129 | E129 | E129 | E129 | E129 | E129 |
| I937 | I937 | I937 | I937 | I937 | I937 | I937 | I937 | I937 | I937 |
| E100 | E100 | E100 | E100 | E100 | E100 | E100 | E100 | E100 | E100 |
| I42627 | I42627 | I42627 | I42627 | I42627 | I42627 | I42627 | I42627 | I42627 | I8614 |
| E222 | E222 | E222 | E222 | E222 | E222 | E222 | E222 | E222 | E452 |
| I6663 | I6663 | I6663 | I6663 | I6663 | | I6663 | I6663 | I6663 | |
| E54 | E54 | E54 | E54 | E54 | I14067 | E54 | E54 | E54 | |
| I7350 | I7350 | I7350 | I7350 | I7350 | | I7350 | I7350 | I7350 | |
| E101 | E101 | E101 | E101 | E101 | E101 | E101 | E101 | E101 | |
| I5704 | I5704 | I5704 | I5704 | I5704 | I5704 | I5704 | I5704 | I5704 | |
| E147 | E147 | E147 | E147 | E147 | E147 | E147 | E147 | E147 | |
| I9950 | I9950 | I9950 | I9950 | I9950 | I9950 | I9950 | I9950 | I9950 | |
| E1222 | E1222 | E1222 | E1222 | E1222 | E1222 | E1222 | E1222 | E1222 | |

$1–10 denote 10 isoforms, arranged from top to bottom as shown in Figure 1B. Exxx and Iyyy represent exons and introns, respectively, where xxx and yyy indicate their lengths. Alternative splicing events are highlighted with a yellow background. $3–6 are APP770-, APP695-, APP751- and APP752-encoding isoforms.

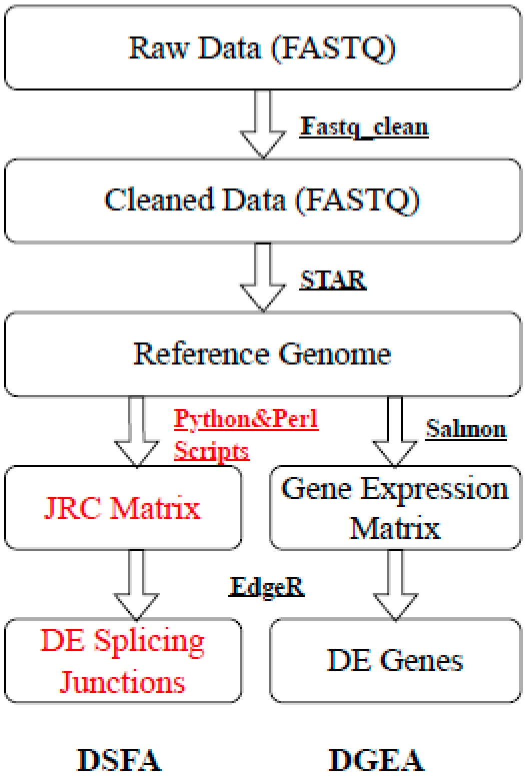


**Figure S1 Workflows of DSFA and DGEA**

To implement differential splicing frequency analysis (DSFA), the Python & Perl scripts have been included in Fastq_clean (https://github.com/gaoshanT/Fastq_clean) to generate a jrc matrix for each dataset using SAM files derived by STAR. the significantly differential expressed (DE) splice junctions and DE genes were selected using the R package edgeR.

Using the Python & Perl scripts, splice junctions can be identified by counting splice-junction (sj) reads that align properly to these junctions, adhering to a set of criteria designed to minimize false positives. These identified splice junctions must satisfy the following criteria: (1) each junction must be covered by at least five junction reads with read lengths of at least 100 bp; (2) each junction read must have an overhang length of at least 5 bp on either side of the junction; and (3) at least one junction read must have an overhang length of at least 10 bp.

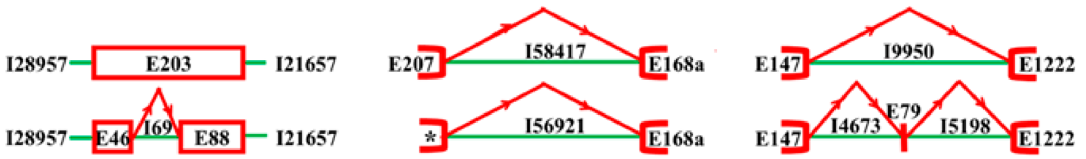


**Figure S2 Three rare splicing junctions**

The exons and introns are indicated in red and green, respectively. Over-expression of U1 snRNA increases the splicing frequencies of rare splicing junctions APP/56921N, APP/5198N, and APP/69N. When the smaller and rare introns are formed in RNA splicing, APP/I58417 is split into APP/I56921 and a novel exon (indicated by *), which encodes a new peptide MLRSCLHDSWARGGCISLRTS. APP/I9950 is split into APP/I4673, APP/E79, and APP/I5198, while APP/E203 is split into APP/E46,

APP/I69, and APP/E88.
